# Effect of codon adaptation on codon-level and gene-level translation efficiency *in vivo*

**DOI:** 10.1186/1471-2164-15-1115

**Published:** 2014-12-16

**Authors:** Kenji Nakahigashi, Yuki Takai, Yuh Shiwa, Mei Wada, Masayuki Honma, Hirofumi Yoshikawa, Masaru Tomita, Akio Kanai, Hirotada Mori

**Affiliations:** Institute for Advanced Biosciences, Keio University, Tsuruoka, Yamagata, 997-0017 Japan; Genome Research Center, NODAI Research Institute, Tokyo University of Agriculture, 1-1-1 Sakuragaoka Setagaya-ku, Tokyo, 156-8502 Japan; Division of Biobank and Data Management, Iwate Tohoku Medical Megabank Organization, Iwate Medical University Disaster Reconstruction Center, 2-1-1 Nishitokuda, Yahaba-cho, Shiwa-gun, Iwate, 028-3694 Japan; Department of Bioscience, Tokyo University of Agriculture, 1-1-1 Sakuragaoka Setagaya-ku, Tokyo, 156-8502 Japan; Systems Biology Program, Graduate School of Media and Governance, Keio University, Fujisawa, 252-8520 Japan; Graduate School of Biological Sciences, Nara Institute of Science and Technology, Ikoma, Nara, 630-0101 Japan

**Keywords:** Ribosome profiling, Codon usage, Codon preference, Chloramphenicol, Translation efficiency

## Abstract

**Background:**

There is a significant difference between synonymous codon usage in many organisms, and it is known that codons used more frequently generally showed efficient decoding rate. At the gene level, however, there are conflicting reports on the existence of a correlation between codon adaptation and translation efficiency, even in the same organism.

**Results:**

To resolve this issue, we cultured *Escherichia coli* under conditions designed to maintain constant levels of mRNA and protein and subjected the cells to ribosome profiling (RP) and mRNA-seq analyses. We showed that the RP results correlated more closely with protein levels generated under similar culture conditions than with the mRNA abundance from the mRNA-seq. Our result indicated that RP/mRNA ratio could be used as a measure of translation efficiency at gene level. On the other hand, the RP data showed that codon-specific ribosome density at the decoding site negatively correlated with codon usage, consistent with the hypothesis that preferred codons display lower ribosome densities due to their faster decoding rate. However, highly codon-adapted genes showed higher ribosome densities at the gene level, indicating that the efficiency of translation initiation, rather than higher elongation efficiency of preferred codons, exerted a greater effect on ribosome density and thus translation efficiency.

**Conclusions:**

These findings indicate that evolutionary pressure on highly expressed genes influenced both codon bias and translation initiation efficiency and therefore explains contradictory findings that codon usage bias correlates with translation efficiency of native genes, but not with the artificially created gene pool, which was not subjected to evolution pressure.

**Electronic supplementary material:**

The online version of this article (doi:10.1186/1471-2164-15-1115) contains supplementary material, which is available to authorized users.

## Background

The amount of each cellular protein is determined by the balance of its translation, degradation, and dilution by cell growth. These factors are typically not balanced due to external and internal changes that affect the levels of protein and mRNA, which in turn lead to discordance between their respective abundances [1, 2]. In a previous study [3], we employed a continuous culture of *Escherichia coli* to make a static cell state where mRNA and protein levels were kept constant [4]. Quantitation of absolute protein and mRNA levels in central carbon metabolism was accomplished by liquid chromatography–tandem mass spectrometry in a multiple reaction monitoring mode and real-time quantitative polymerase chain reaction (PCR) methods, respectively [3]. Although the levels of protein and mRNA were constant, and most proteins were stable in this condition, correlation between quantified protein and mRNA levels remained moderate (correlation coefficient 0.56–0.58), indicating limited accuracy of the quantification techniques or a difference in translation efficiencies.

Many factors affect the translation efficiency of an mRNA [5], and among them, mRNA folding around the translation initiation site and codon usage bias are the most studied. Evidence indicates that the rate of translation initiation is influenced by mRNA folding around the start codon and ribosome binding site (RBS), and systematic analyses of the expression of synthetic genes indicate that this factor most strongly influences translation efficiency [6–8]. In contrast, codon usage bias is considered to influence the elongation rate because each codon is decoded at a different rate [9, 10]. When initiation is limiting without considering ribosome collisions or mRNA degradation, elongation should not affect translation efficiency [11, 12]. However, data acquired from genome-wide analysis of protein and mRNA levels of endogenous genes show that codon bias correlates with translation efficiency [13, 14].

As an alternative to comparing protein and mRNA abundances directly, the translational efficiency of mRNA was analyzed by measuring the amount of ribosomes bound to mRNA [15, 16]. Ribosome density, i.e., the number of ribosomes bound per unit mRNA length is affected mainly by two factors, namely initiation rate and elongation rate. It increases as a function of higher initiation rates and, decreases as a function of increased elongation rate. Hence, it is not possible to precisely derive translation efficiency from ribosome density. However, considering that initiation is generally rate limiting, such predictions have been made [11, 12].

Because quantification of the abundances of mRNA and ribosome-bound mRNAs can be performed using the same principle with some modification of the RNA isolation method [15–17], comparing the two values can be more accurate than comparing mRNA abundance data with protein abundance data, which is obtained using very different techniques and tend to have larger errors and lower coverage. More recently, advances in high-performance sequencing enable investigators to analyze transcripts with increased coverage, higher dynamic range, and greater resolution. These techniques make it possible to perform ribosome density mapping at the nucleotide level, which is called ribosome profiling (RP) [17, 18].

In the present study, we performed RP and mRNA-seq analysis in glucose-limited continuous culture of *E. coli* that maintains a constant level of each mRNA and its translation efficiency. We determined gene-level ribosome density and also quantified the differences in ribosome density between codons located at the ribosomal A-site and nearby positions. In the specified condition, ribosome density at A-site showed negative correlation with codon usage and decoding speed. Thus, lower ribosome densities of genes with highly adapted codon usage were anticipated. However, we found that the ribosome densities for such genes increased, indicating that the initiation rate rather than the elongation rate was the major contributor to the ribosome density and translation efficiency of highly codon-adapted genes.

## Results

### Comparison of transcripts and ribosome profiling at the gene level

**Figure 1:**
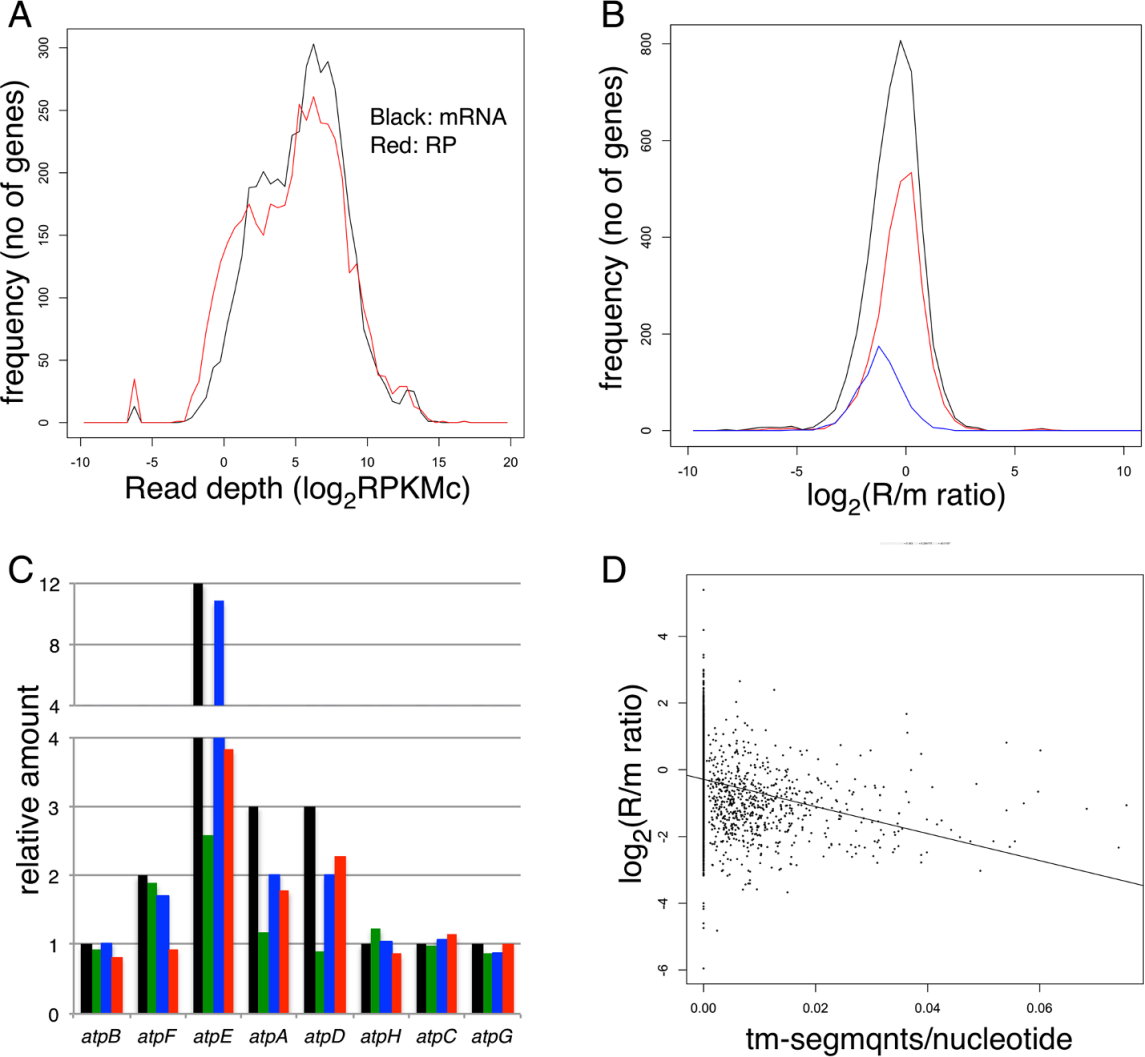
**Read depth and R/m ratio of genes and their relation to protein abundance and membrane association. (A)** Histogram of read depths of coding genes. Black line, mRNA-seq data; red line, RP data. **(B)** Histogram of the R/m ratio of all (4331) genes (black) or genes classified according to the cellular location of their products: 2507 cytoplasmic (red) and 762 integral membrane proteins (blue). **(C)** Read depth and R/m ratio of the *atp* operon compared with the subunit composition of F0/F1 ATPase. Subunit composition (black), mRNA-seq (green), RP (blue), and R/m ratio (red). Relative values with the average value of genes encoding one-subunit per complex protein (*atpB*, *H*, *C*, and *G*). **(D)** R/m ratio and frequency of transmembrane segments. The number of transmembrane segments in the protein encoded by each gene was divided by the ORF length and plotted against the R/m ratio of the gene. Spearman correlation coefficient = -0.363, x-intercept = -0.29, slope = -40.5.

To compare mRNA-seq and ribosomal profiling (RP) data at the gene level, the sequence reads taken from the glucose-limited continuous culture were mapped to the *E. coli* genome. The read counts (depth) mapped to a gene region was first normalized between samples according to the total read numbers mapped to coding sequences (CDS) reads and then according to gene length. The resulted read per kilobase million CDS reads (RPKMc) was used as the normalized depth for the gene. The distribution of RPKMc for mRNA-seq and RP [calculated as the average of three or four samples, respectively, (Figure 1A)] mainly comprised two large peaks, and the distribution of RP was slightly wider (80% of the genes, excluding 10% from both ends within 200- and 500-fold differences for mRNA-seq and RP, respectively). In contrast, plots of the ratio of RP to mRNA-seq (R/m ratio) show a sharper peak with a seven-fold difference between 80% of the genes (Figure 1B), indicating that the difference in ribosome density was considerably smaller compared with the difference in the amount of mRNA.

We reported the quantification of 55 central carbon enzymes and their cognate mRNAs under the glucose-limited continuous culture [3]. However, there was a moderate correlation between the levels of proteins and their cognate mRNAs (correlation coefficients of 0.56 and 0.58 for dilution rates of 0.7 h^-1^ or 0.5 h^-1^, respectively).

Because the condition employed in this study (glucose-limited continuous culture at D = 0.6 h^-1^) was considerably similar to the previous work, we compared the expression data from mRNA-seq and RP with the corresponding protein abundance from the previous study. The mRNA abundance determined using mRNA-seq correlated more closely (correlation coefficient = 0.63) with protein abundances at 0.5 and 0.7 h^-1^ compared with the previous mRNA abundance data determined using RT-PCR, indicating that mRNA-seq generates more accurate data for comparison between genes. However, the depth determined using RP showed a considerably increased correlation with protein abundance (correlation coefficients = 0.80 and 0.77 at 0.7 and 0.5 h^-1^, respectively, Additional file 1: Figure S1 and Table S1).

**Figure 2:**
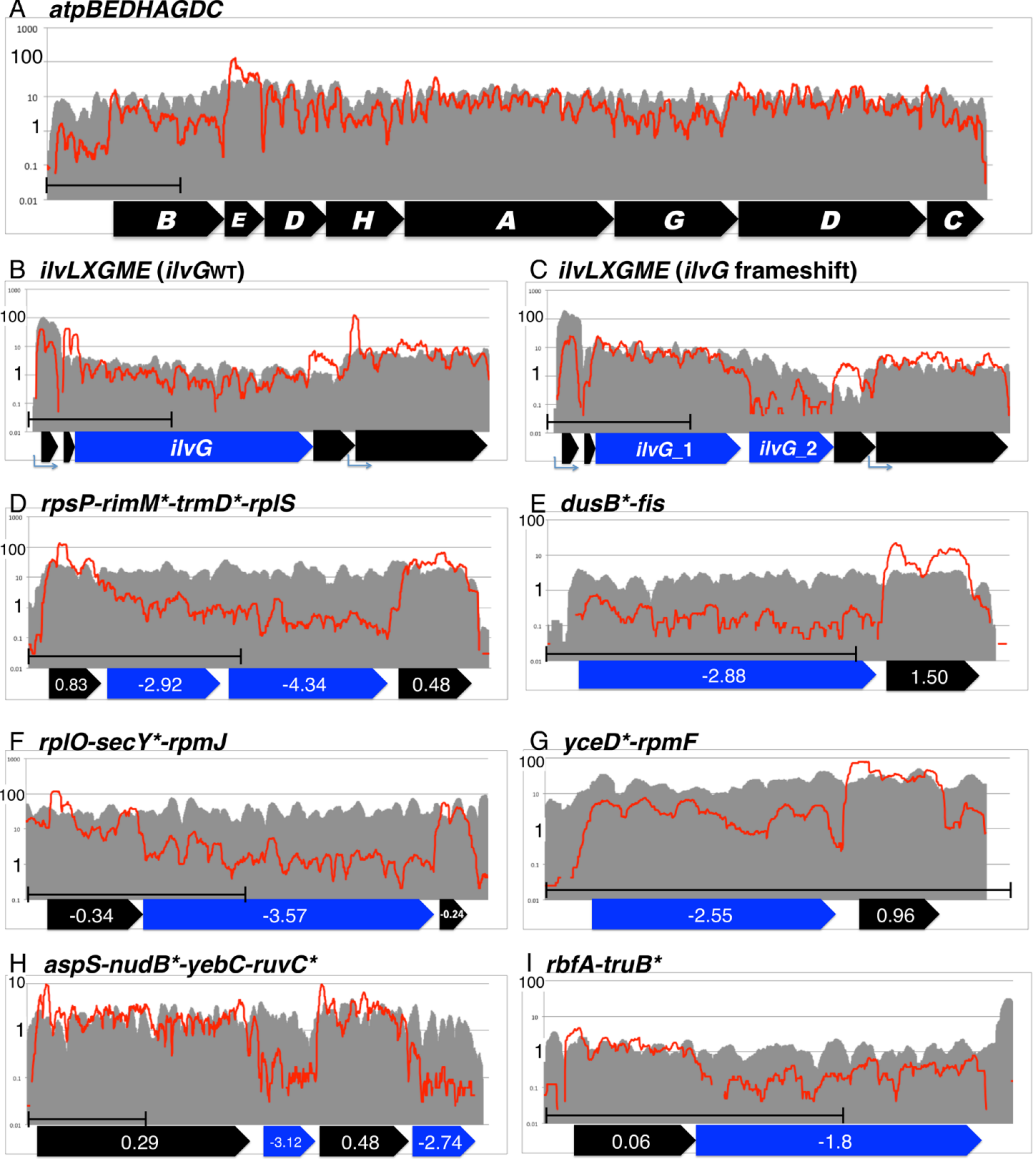
**mRNA and RP depths in differentially translated operons.** The mRNA and RP depths of each genome position are shown as gray area and red line, respectively, on a 1-kb scale. The position of each gene is shown as a thick arrow below **(A)**. The *ilvG* gene with or without a frameshift mutation is highlighted in blue (**B** and **C**, respectively). The R/m ratio of each gene is shown inside arrows **(D–I)**, and genes with low R/m ratios are marked with an asterisk next to their name and shown in blue.

Next, we analyzed the results for the F0/F1 ATPase protein complex whose subunit composition, i.e., relative protein abundance was well studied [19, 20]. In the *atp* operon, the structural genes encoding F0/F1 ATPase (*atpBEFHAGD*) are transcribed as a single mRNA, but the resulting protein forms a complex consisting of different numbers of each subunit [21]. Figure 1C shows the relative RPKMc (green: mRNA, blue: RP) and the R/m ratio (red) of each gene, along with the subunit composition in a complex (black). The depth of mRNA and RP along the operon are shown in Figure 2A. As recently shown by Li et al. [22], RP depth (blue) most closely correlated with subunit composition (black) where depth of *atpE* (c subunit, 10–12 per complex) was greater than ten-fold higher compared with depth of *atpB, H, C,* and *G* (one per complex), and the depth of *atpF, A,* and *D* (2, 3, 3 per complex) was slightly higher compared with the four subunits present as one per complex. However, mRNA depth and R/m ratio also showed positive correlation with the subunit composition. These results indicate that RP depth more precisely indicates the amount of protein synthesis compared with mRNA depth. The reason that the mRNA depth showed a similar difference between the genes with the RP depth may be explained by protective effect of ribosomes from mRNA degradation, rho-dependent termination, or both [23]. One possible example of the protective effect against the 5′–3′ nuclease is the mRNA depth within the *atpB* coding region, which gradually increased toward the 3′ terminus to the adjacent high-density *atpE* (Figure 2A). It is likely that this pattern was caused by the low ribosome density of the *atpB* coding region compared to that of the *atpE* coding region, which resulted in a higher degradation rate of the former.

To see the effect of translation on the mRNA amount more clearly, result around *ilvG* gene, which was split into *ilvG*_1 and *ilvG*_2 by a frame-shift mutation in the strain used, was compared with the result of the strain, which has intact *ilvG*. In the intact *ilvG* (*ilvG*_wt_) strain (Figure 2B), RP and mRNA remained constant until the end of gene. In contrast, RP reads dropped sharply after the frameshift mutation and did not recover at the start of the *ilvG*_2 ORF in the *ilvG* frameshifted strain (Figure 2C). Moreover, the depth of mRNA dropped gradually after the frameshift, indicating an mRNA protective effect of the ribosome.

Even though these results covered a limited number of genes, they showed that the depth of RP serves as a better proxy for protein abundance compared with mRNA abundance, as described previously [18], and that the R/m ratio, though not exactly, reflects translation efficiency.

### Mixed function operons composed of differently translated genes

The results described above prompted us to find genes with abnormal ribosome densities as candidate examples of abnormal translation efficiency. Significant analysis of microarray (SAM) [24] was used to find genes with significantly different mRNA-seq and RP depths. We selected 161 of such genes (false-positive rate = 0.000 at a distance of 8.5) and used them for the enrichment analysis. Enriched categories of gene classification within the selected gene were identified using DAVID [25]. Because, in this initial analysis, many of the enriched categories were related to the membrane, the R/m ratio of genes encoding cytoplasmic and integral membrane proteins [26] was analyzed separately (Figure 1B). As we expected, the R/m ratio of inner membrane proteins was about 1.8-fold lower compared with those of cytoplasmic proteins. To test the relation of membrane affinity of the proteins and the R/m ratios, the number of transmembrane segments per protein length was compared according to the R/m ratio (Figure 1D). These results show that proteins with greater membrane segment frequencies, which should exhibit a stronger affinity to the membrane, had lower R/m ratios and the affinity of the nascent peptide to the membrane may have affected translational efficiency. However, another possibility is that the lower R/m ratio is due to the low yield of ribosomes while the nascent peptide is incorporated in the membrane.

To avoid this effect, genes coding for cytoplasmic protein were used for the SAM selection and 99 genes (15 high and 84 low ribosome density) were selected (Additional file 1: Table S2) and subjected to the enrichment analysis. Within the five categories enriched (P < 0.05, Table 1), four (homologous recombination, ribosome, ncRNA metabolic process, DNA replication, and tRNA aminoacylation) were related to basic processes for transmitting and expressing genetic information. Further, three of the four genes in the remaining category of pyrimidine metabolism including *holA* and *dnaE* (genes encoding DNA polymerase III subunits) and *rpoA* (encoding an RNA polymerase subunit) were related to these processes. This result indicates the possibility that genes for such functions may use the difference in initiation rate to produce different amounts of proteins encoded in the same operon.

**Table 1:** **Enriched categories in the genes with abnormal R/m ratio**

Term ^*1^	P Value ^*2^	Gene ^*3^
Homologous recombination^*4^	1.90 × 10^-04^	*ruvC, recB, holA, dnaE*
Ribosome^*5^	1.70 × 10^-05^	*rplY, rpsR, rplI, asnC, rpsF, rimK*
ncRNA metabolic process^*6^	2.50 × 10^-04^	*ygfZ, yadB, truB, truA, trmD, rimM, dusB, asnC*
DNA replication^*7^	2.30 × 10^-03^	*holA, dnaE, dnaG*
Pyrimidine metabolism^*8^	1.40 × 10^-03^	*pyrC, holA, rpoA, dnaE*
tRNA aminoacylation^*9^	2.90 × 10^-02^	*yadB, asnC, truA*

*1:The term enriched, *2:Pvalue of the term selected, *3:name of the genes within the selected term, *4:GO:0004518, *5:GO:0005840, *6:GO:0034660, *7:ecl03030 (KEGG pathway), *8:ecl00240 (KEGG pathway), *9:GO:0043039.

The rpsP-rimM*-trmD*-rplS operon provides a known example of such an operon, where two nonribosomal proteins encoded within the operon are translated less efficiently compared with the two ribosomal proteins located at 5′ and 3′ ends of the operon [27] (Genes marked by * were selected and assigned to the five enriched categories). Accordingly, while the depth of the mRNA reads did not change throughout the operon, RP depths dropped by ten-fold for most of the rimM* and trmD* coding regions compared with those of *rpsP* and *rplS* (Figure 2D), indicating that low translation efficiency is associated with lower ribosome density. The dusB*-fis, rplM-secY*-rpmJ, and yceD*-rpmF operons (Figure 2E–G) provide similar examples. Thus, genes encoding abundant protein, such as nucleoid or ribosomal protein, form an operon with genes for translational function required for less amount, or in case of yceD*, genes without 5′-noncoding leader and translate less efficiently [28]. The RbfA-truB* and aspS-nudB*-yebV-ruvC* operons also serve as examples of mixed-function operons encoding differentially translated genes (Figure 2H, I).

Such an operon with genes of different translational efficiencies is a logical strategy in basic gene function for attaining different requirement of protein level, while keeping the same tight control of transcriptional regulation.

### R/m ratio and other indices associated with gene expression

**Figure 3:**
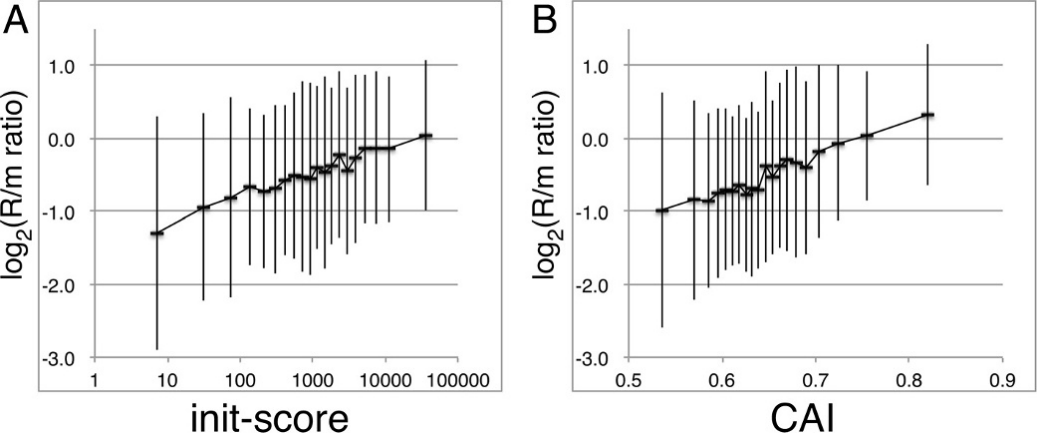
**Relation of the R/m ratio to the init-score or CAI.** All genes were classified into 20 groups according to the init-score **(A)** or CAI **(B)**, and the average ± SD of the R/m ratio of genes within each group is indicated.

mRNA folding around the start codon and ribosome binding site (RBS) influence translation efficiency at the level of initiation, and shown as a major factor by tests of the translation of artificial gene sets encoding the same green fluorescent protein (GFP) [7, 29, 30]. We tested the effect of initiation efficiency on the R/m ratio. For this, we allocated the genes into 20 groups depending of their predicted initiation rate (init-score, predicted by their RBS sequences and secondary structures [30]) from highest to lowest, and the average R/m ratio within each group was plotted as a function of the average init-score of the group (Figure 3A). As we expected, groups with higher init-score had higher R/m ratios, and the difference between the highest and lowest groups was approximately 2.2-fold. The deviation within the group was large, but the initiation strength and R/m ratio of each gene correlated significantly (tau = 1.69, P < 2.2 × 10^-16^) according to the results of Kendall’s rank correlation test. Considering that the R/m ratios of 80% of genes fell within a range of seven-fold, this difference indicates that the R/m ratio correlates with the predicted translation efficiency of mRNAs. When the R/m ratio was compared with the affinity of RBS for the anti-RBS sequence of 16S rRNA, no such clear correlation was detected (data not shown), indicating that the strength of this affinity alone did not alter translation efficiencies, consistent with the results of another study [30].

Codon usage is also considered to affect translation efficiency, and although studies using libraries encoding GFP argue against this hypothesis [7, 29, 30], the comparison studies measuring endogenous protein and genes favor it [13, 14]. The codon adaptation index (CAI) [31] is a score calculated from the codon usage of highly expressed genes and is known to show positive correlation with transcription level. Similar positive correlation of CAI and translation efficiency, deduced from protein and mRNA levels is found and is explained by the elongation rate [13]. When we determined the correlation of R/m ratio with CAI, using the same method used for init-score, a similar positive correlation was observed (Figure 3B), and the difference between the highest and lowest group was 2.1-fold. In addition, CAI and R/m ratio of each gene correlated significantly (tau = 1.88, P < 2.2 × 10^-16^) according to the results of Kendall’s rank correlation test. This result indicates that initiation efficiency is significantly higher for genes with higher codon adaptation.

### Precise position of the ribosome on mRNA

**Figure 4:**
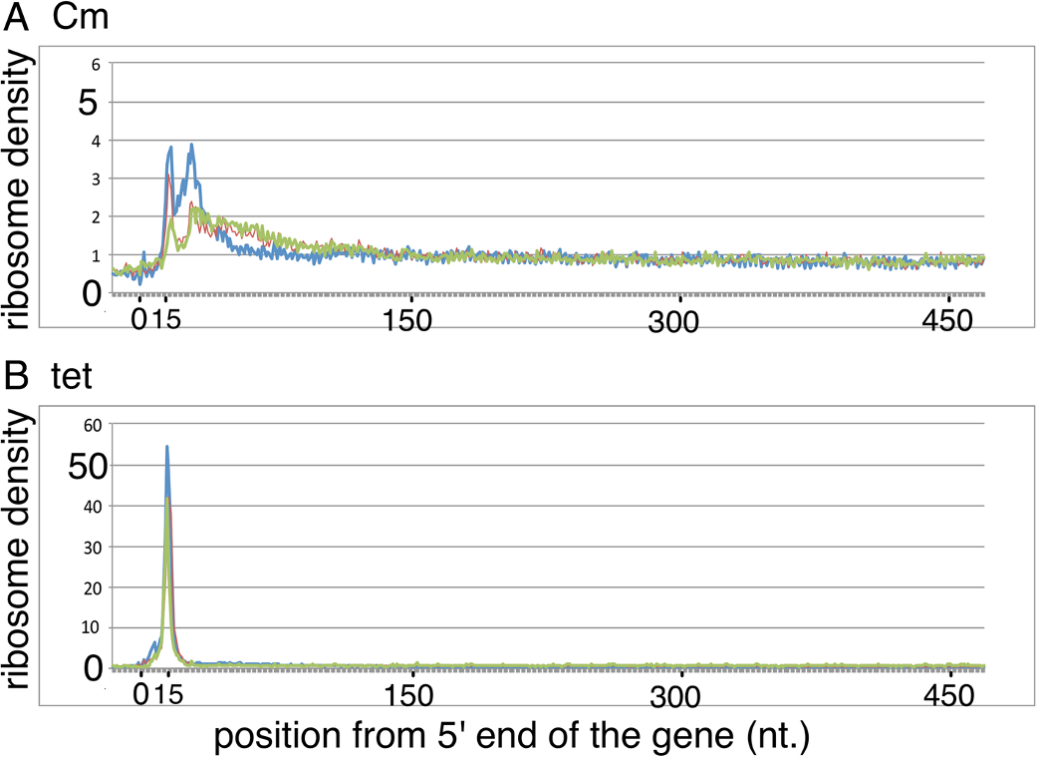
**Average ribosome depth of genes mapped using 3′ end reads.** Genes longer than 500 bp were selected, and ribosome depths, from -20 to +480, from the 5′ end of the gene, were normalized to an average value of one, then the depths in all genes were averaged. The ribosome depth calculated using 3′ end of sequence reads were used and the samples treated with Cm **(A)** or Tet **(B)** to inhibit translation was shown.

Determining the position of the ribosome bound to mRNA at high resolution is possible using RP combined with high-throughput sequencing [17]. For this purpose, we first tested the suitability of using either the 5′ or 3′ end of each sequence read for mapping of the ribosome position. The results show that using the 3′ end of the sequence read for mapping average ribosome density of ORF, a sharp peak, 15 nucleotides (nt) upstream from the first position of the initiation codon, and a broader peak, at 5–30 nt downstream, is observed (Figure 4A). Such a pattern was also observed in other studies of *E. coli* and yeast [17, 32]. Because using the 5′ end of sequence read did not show such clear peaks (data not shown), we used 3′ end for precise mapping. Probably RNaseI cleaves precisely 3′ to the mRNA protected region of the ribosome, while the position that is cleaved 5′ to the protected region is more variable.

Inspection of the pattern shown in Figure 4A suggests that the first sharp peak corresponded to the initiation complex where the initiation codon is located at the peptidyl site (P-site). Therefore, RNaseI cleaves approximately 12 nucleotides (nts) downstream from the first base of the aminoacyl site (A-site) codon. This notion was confirmed by the signal from the ribosome arrest position of *secM* [33] (data not shown) and the evidence described below. Here after, we count the first base of the A-site as position 0, and 5′ direction as negative.To test whether these results were due to the intrinsic nature of translation or due to the chloramphenicol (Cm) used to stop translation during and after harvesting the cells, the same experiments were performed using tetracycline (Tet) as translation inhibitor instead of Cm (Figure 4B). Notably, the pattern at the 5′ terminal region differed from that using Cm because the peak corresponding to the initiation codon at the P-site was much larger, accounting for nearly half of the CDS reads, and a second peak was not observed. For this reason, we decided to use both Cm and Tet-treated results for the precise mapping and excluded the first 20 and last 10 codons of the gene from the analysis.

### Difference in ribosome density between codons

**Figure 5:**
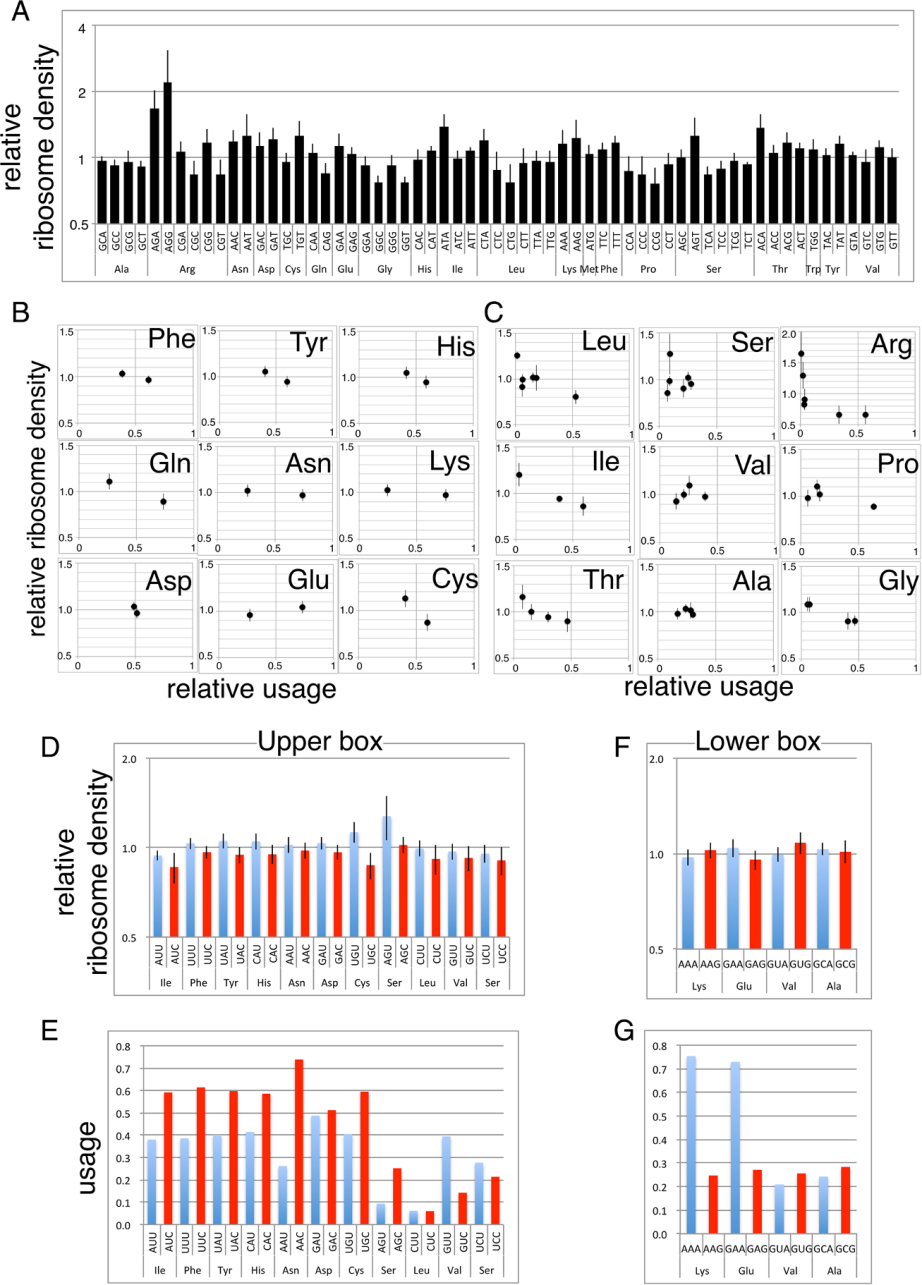
**A-site ribosome density at each codon and its relation to codon use. (A)** The A-site ribosome density of the 61 coding codons (average ± SD of three samples each from cultures treated with Cm or Tet). **(B–G)** The ribosome density shown in **(A)** was modified as average equal to one within each amino acid codon and compared with relative use at the transcript level (total use of each codon was defined = 1). **(B, C)** Comparison of use and ribosome density of two codon families **(B)** and multicodon families **(C)**. **(D–G)** Relative ribosome density **(D)**, **(F)** and use **(E)**, **(G)** of codon pairs decoded by a single tRNA. The pair of codons shown in blue and red share one tRNA for decoding.

We did not expect higher R/m ratios for genes with high CAI. Because the expected higher translation efficiencies of these genes is due to increased elongation rates, and if the initiation rate is the same, the increased elongation rate should decrease ribosome density. To address this question, we compared the ribosome density of each codon when it is located at the A-site. The relative ribosome density at a specific position was calculated by dividing the read depth of the position by the average depth of 121 bases (60 nts to the 5′ and 3′ directions), and the average depth of each codon located at the A-site (position 0) was calculated using all CDS reads. We used three samples from each of cultures treated with Cm or Tet, and calculated average depth ± standard deviation of each codon (Figure 5A). As expected, we found that the ribosome densities of pronounced rare codons (Arg-AGA and AGG) were the highest, and those of other rare codons, Ile-AUA and Leu-CUA, were also significantly higher than their synonymous codons (P < 0.05, Wilcoxon test).Therefore, we conducted further tests to determine the relation of synonymous codon usage to A-site ribosome density. Synonymous codon usage at the mRNA level was calculated from mRNA-seq data as the sum of a particular codon divided by the sum of all codons that encode the same amino acid. We compared the resulting codon usage in each amino acid with the A-site ribosome density. In two-codon family amino acids (Figure 5B), the ribosome densities of seven of nine were lower at the preferred codon (three were significant at P < 0.05, Wilcoxon test). In one exception (Asp), the use and ribosome density of two codons (GAU and GAC) were very close, and the other exception (Glu) will be discussed below. In multicodon families (Figure 5C), Leu, Ile, Thr, Arg and Gly, codon usage and A-site density were in good negative correlation. There was no clear correlation for other multicodon amino acids including Ser, Val, Ala, and Pro; however, in these cases, deviation of codon usage was lower than those with negative correlations.

We next compared the codon-level ribosome density with another report of the in vivo decoding speed. Curran and Yarus used the rate of frameshifts versus normal decoding (R_tRNA_/R_shift_) to obtain the translation efficiency of 29 codons [10]. Except for four Pro codons, R_tRNA_/R_shift_ correlated inversely with the present ribosome density data (Additional file 1: Figure S2), indicating the consistency of the two methods.

The different decoding efficiency between codons is attributed, in part, to the difference in the amount of tRNA pool [34], and recognition by the same tRNA species may contribute as well. This effect was tested by comparing NNU and NNC codon pairs in a 2/2 split box, which is translated by a single tRNA with a GXX anticodon (Figure 5D, E). Although, the difference of ribosome density in each pair was small and only one of them (Cys) showed significant difference (Wilcoxon signed rank, p < 0.05), density at NNC was lower than the paired NNU codon in all the 11 codon-pairs, indicating significant higher ribosome density at NNU than at NNC codon (p < 0.001 by the Wilcoxon signed rank). In accordance with that, NNU codon showed lower usage than the paired NNC codon in 9 of the 11 pairs, indicating the tendency of high usage of efficient codon of the pair.In contrast, for many NNA and NNG codon pairs, there are tRNAs with UXX and CXX anticodons and decoding of the two codons is affected by both factors. To avoid this, four pairs decoded by a single UXX anticodon tRNA were analyzed. Unlike the NNU and NNC pairs, we found two cases each with an NNG or NNA codon with a higher ribosome density (Figure 5F). Nevertheless, Lys-AAA and Glu-GAA gained strong preference compared with the corresponding AAG and GAG codons (Figure 5G), which cannot be explained by the decoding efficiency at A-site.

### Codon-level difference in the ribosome density at other positions

**Figure 6:**
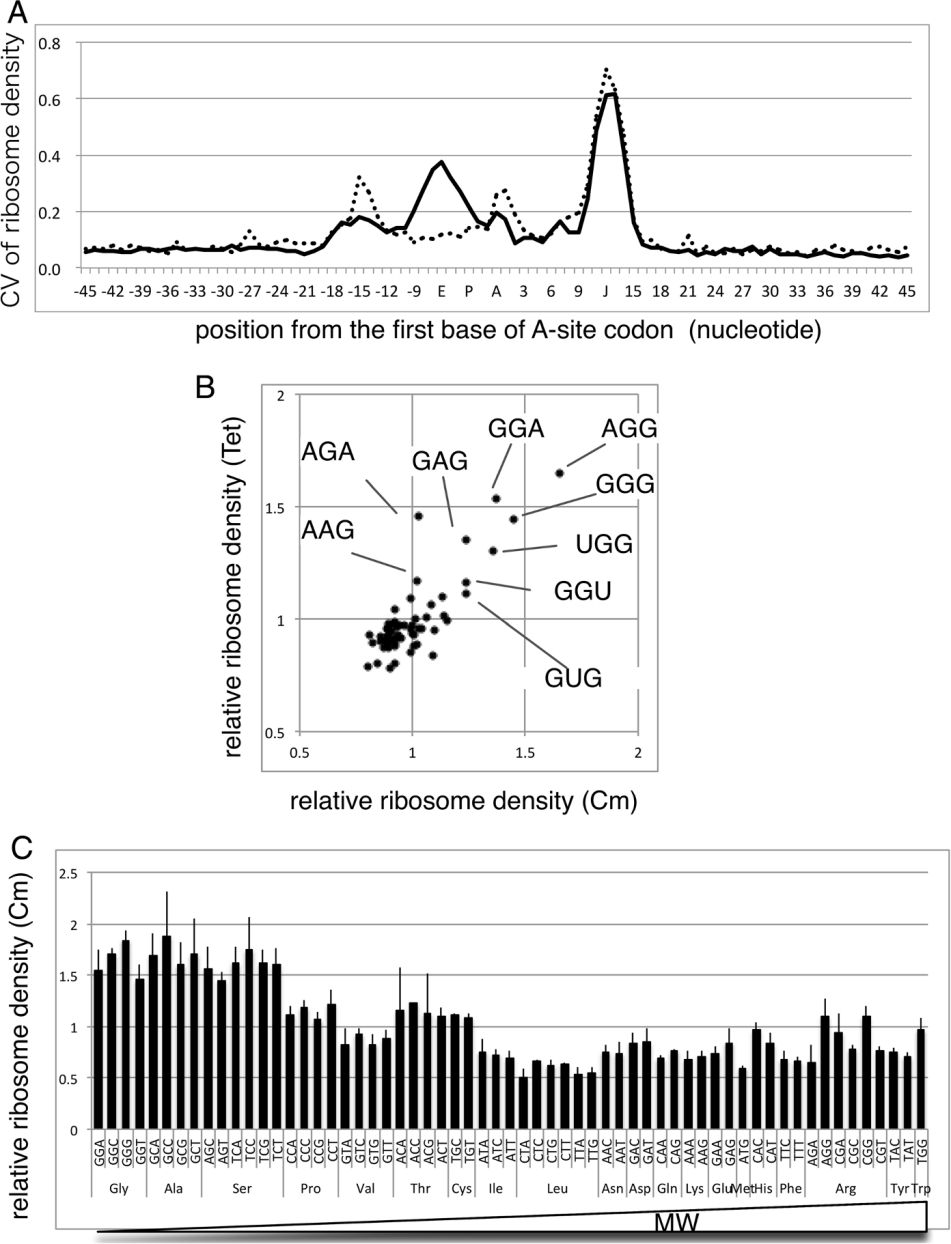
**Local structures affecting ribosome density. A)** Coefficient of variation of ribosome densities of 61 codons located from -45 to +45 nts. Positions relative to the A-site are displayed along the horizontal axis, and the corresponding ribosomal A-, P-, and E-site are designated A, P, and E, and the read-end position is indicated by the J. Solid line: Cm, Dotted line: Tet. **(B)** High ribosome density of G-rich triplets located from -15 to -18 positions. Relative ribosome densities of triplets in samples from cultures treated with Cm or Tet that showed the highest similarity (-16 for Cm and -17 for Tet) are shown, and triplets with high densities are marked. **(C)** The relative ribosome densities of codons at the E-site (-6) of samples taken from cultures treated with Cm (average ± SD) are aligned according to the molecular weight of encoded amino acid side chain.

The difference in ribosome density at the A-site codon prompted us to test the difference of ribosome density between codons when they were located the positions around A-site. Therefore, we calculated the ribosome density of each codon at each position; to show the possible effect of antibiotics used, samples treated with Cm and Tet were summarized separately (Additional file 1: Figure S3). To reveal the position where each of the codons generates different ribosome densities, the standard deviation of ribosome densities of 61 codon species at each position were calculated (Figure 6A).

The highest peak near position +12 (denoted J), corresponding to the end of the mRNA fragment, should be due to differences in cloning efficiency of the 3′ terminal sequence, but is not associated with translation. The peak near position 0 (A-site) corresponds to the difference in the A-site ribosome density described above. After the A-site, the deviation decreases and then increases near the exit site (E-site, position *-*6) only in samples treated with Cm. Then, another peak common to samples treated with Cm and Tet is present near position -15. To investigate whether the patterns of samples from cultures treated with Cm or Tet were similar at this -15 region, the depths of each codon were compared (Figure 6B). As we expected, they showed similar patterns of G-rich, but no C, codons with high ribosome density. Li et al. [35] detected an anti-Shine–Dalgarno (SD)-like sequence in the coding region where translation pauses, and because these G-rich triplets were anti-SD-like, we interpret that the difference in ribosome density at these positions is due to the effect of anti-SD-like sequence. This effect may explain the infrequent use of Glu-GAG and Lys-AAG codons because the ribosome densities were significantly higher than those of GAA and AAA codons, respectively, while the difference at the A-site was smaller.In contrast, the variation in ribosome densities of Cm-treated samples around the E-site may be associated with an effect of Cm. Ribosome density was the highest when the amino acid coded by the E-site codon (E-site amino acid) was very small (Gly, Ala, and Ser), and the next highest group (Pro, Thr, and Cys) also had small side chains (Figure 6C). This systematic difference indicated that the second position of nascent peptide corresponding to the E-site amino acid, not the codon, caused this difference.

## Discussion

The results from the gene-level comparison of RP and mRNA-seq data with protein abundance indicated that RP reflected protein abundance more efficiently than mRNA-seq. Moreover, this analysis shows that the translation initiation rate significantly affected translation efficiency, consistent with the findings of others [11, 12], and that ratio of RP and mRNA-seq (R/m ratio) can be used to estimate the translation efficiency of genes.

Utilizing the R/m ratio we showed that genes for fundamental biological process such as translation, RNA modification, and DNA replication/repair or recombination were significantly enriched. These genes are frequently present in mixed-function operons, and the difference in translation rate should serve as a very efficient method to synthesize different amounts of proteins while maintaining the operon’s stringent transcriptional regulation. Moreover, we discovered a positive correlation between the R/m ratio with both the efficiency of translation initiation predicted from 5′ terminal sequence [29] and the CAI [31], indicating that these factors affect the ribosome density of endogenious *E. coli* genes (Figure 3A, B). We expected the former finding, because a high initiation rate should increase ribosome density. In contrast, the latter finding was not expected because a high CAI might increase translation by increasing the elongation rate, and thus decrease ribosome density.

In contrast, ribosome density at the codon level was consistent with the hypothesis that an increased elongation rate decreases ribosome density because the relative ribosome depth at the A-site generally reflects codon preference and decoding efficiency. Thus, the observed difference in the R/m ratio according to CAI should have represented the combined results of differences in the elongation and initiation rates, and the latter had a larger effect. Although correlation of CAI with mRNA abundance is universally conserved in many organisms, codon adaptation is not directly connected to transcription level, and evolutional pressure for high expression genes should have favored mutations leading to highly codon-adapted genes [12, 31, 36]. It is plausible to think that the evolutionary pressure should also favor mutations that cause high initiation rate and more effectively affect the initiation rate of the same set of high expression genes, because small numbers of mutations are enough to change the initiation rate rather than changing the codon bias of entire coding region.

This result also agrees with the apparently conflicting results that extent of codon bias is not correlated with the translation efficiency of the sets of artificial genes [6, 7] that had not experienced evolutionary pressure, but the codon bias shows positive correlation with the protein/mRNA ratio of endogenous proteins [13, 14].

As the codon-level difference of A-site ribosome density was three-fold at the most and the frequency of rare codons was low, the difference of average ribosome density between genes caused by this effect was very small (the maximum difference between the highest and lowest genes was 1.21-fold, and 1.08-fold for 90% of the genes, when calculated from the codon composition of each gene and the ribosome density of each codon). The difference in ribosome density between codons determined here was smaller than the difference in the decoding rate reported by others [9, 10], and it is possible that the resolution of our analysis was not sufficient to show absolute difference between adjacent codons. If our values are adjusted to match the data of Curran and Yarus [10], the difference between high and low codons become 133-fold. In this case, the maximum difference between genes is 2.07-fold and 1.33-fold for 90% of the genes, which will increase the difference in the translational efficiency of high and low adapted genes significantly; however, still the contribution of the initiation efficiency is considerably larger than the elongation efficiency.

Li et al. reported that an anti-Shine–Dalgarno-like sequence within coding sequence causes translational pausing [35], and we interpret that the high ribosome density of G-rich codons observed around the -15 position (Figure 6B) should be responsible for this. The property may explain the slower translation rate in vivo of the Gln-GAG codon compared with the Gln-GAA codon [37] although the two codons share the same tRNA. The ribosome density of GAG was lower than that of GAA at the A-site, but at the -15 position, the ribosome density of GAG was much higher than that of GAA codon at the same position, and the decoding speed determined using a lacZ-fusion system [37] should be affected at both positions. Further, the decreased use of GAG compared with GAA would indicate that the use of the cognate codon evolved according to the overall efficiency of translation, as reported by Li et al. [35].

Another notable finding of the present study is that local ribosome density around the E-site was different between Cm and Tet treated samples. Both antibiotics inhibit translation elongation although, Tet prevents the stable binding of tRNA to the ribosome by directly overlapping with the anticodon stem-loop of tRNA [38], and there are no reports of codon dependence. In contrast, Cm inhibits protein biosynthesis by targeting the peptidyl transferase center on the large ribosomal subunit, and structural analyses show that Cm binds to the A-site of the peptidyl transferase center (PTC) [39, 40]. However, another binding site was also suggested and binding to the hydrophobic crevice at the entrance to the exit tunnel was revealed by structural analysis of Cm bound to archaeal ribosomes as well as by biochemical analysis of the eubacterial ribosome [41, 42]. Further, template mRNA-sequence dependency in inhibitory effect of Cm is reported. For example, Kucan and Lipmann reported strong inhibition by Cm of poly(UC)- and poly(UG)-directed polypeptide synthesis and weaker inhibition of poly(U)- and poly(UA)-directed polypeptide synthesis [43]. Because poly(UC) encodes Ser, Phe, Pro, and Leu and poly(UG) encodes Cys, Tyr, Leu, Gly, and Val, strong inhibition by Ser and Gly at the E-site is expected for these templates by our results. Instead, none of the amino acids coded by poly(U) or poly(UA) generate a strong peak at the E-site in Cm-treated samples. Thus, the sequence dependency reported by Kucan and Lipmann may not have arisen by binding to PTC, but instead, by the action of Cm bound to the second binding site at the entrance to the exit tunnel where the E-site amino acid resides.

## Conclusions

We applied ribosome profiling and mRNA-seq techniques at the gene-level and codon-level analysis of the translation efficiency in *E. coli* and showed that genes with high codon adaptation shows higher translation initiation efficiency, as well as higher translation elongation efficiencies. The former effect exerted more impact on their ribosome density and translational efficiency. In addition, gene-level analysis showed that the differences in translation efficiency were used to attain demand for different protein amount while keeping the tight transcriptional control of them within a single mRNA. Moreover, codon-level analysis revealed not only the intrinsic nature of the translational apparatus but also differences caused by antibiotic treatment, which were probably impacted by the spectrum of antibiotics.

## Methods

### Bacterial strains

The BW25113 (*lacI*^q^*rrnB*_T14_ Δ*lacZ*_WJ16_*hsdR514* Δ*araBAD*_AH33_ Δ*rhaBAD*_LD78_*ilvG rph*_1_) strain of *E. coli* [44] was used for most experiments. The *smpB* deletion mutant of BW25113 [45] was also used, and the data were combined with those acquired using the wild-type strain because the data were essentially the same. An *ilvG-*reverted derivative of MG1655 (*ilvG rfb*_50_*rph*_1_) [46] harboring a frameshift at codon 327 (Ile) was reverted by inserting AT [47], and used for analysis of the *ilvLXGME* operon.

### Culture method

Glucose-limited chemostat cultures were grown using 1 l of synthetic MOPS medium [48] containing 0.4% glucose and 19 mM NH_4_Cl. Cultures were grown aerobically at 37°C in a 2-l jar fermentor (BMJ-PI; Able, Tokyo, Japan) equipped with sensors for pH, dissolved oxygen concentration, and temperature. The flow rate of air was maintained at 1 l/min, and pH 7.0 was maintained by automatic addition of 5 N HCl or 2 N NaOH during the culture. The dilution rate was 0.6 h^-1^.

### RNA extraction

RNA was extracted from 12 ml of the culture taken directly from the jar, which was mixed immediately with 0.5 volume of 2% SDS, 16 mM EDTA, heated to 100°C, placed in a bath containing boiling water for 5 min, and mixed with an equal volume of phenol/chloroform, pH 5.2 (Ambion, Waltham, MA, USA). After further purification using phenol/chloroform and chloroform, nucleic acids were precipitated with ethanol. To construct an mRNA library, rRNA was removed using a microbeEXPRESS rRNA removal kit (Ambion), and small RNAs including 5S and tRNAs were removed using an RNeasy column (Qiagen, Venlo, Netherlands). The mRNA-enriched fraction was fragmented using RNA Fragmentation Reagents (Ambion), and 30–70 nt fragments were purified using acrylamide gel electrophoresis under denaturing conditions.

### Ribosome isolation

Immediately after harvesting culture samples for RNA purification or at least 30 min later, polysomes were isolated as essentially described by Maki [49]. Cm or Tet (100 μg/ml or 40 μg/ml, final concentrations, respectively) was added, and after 30 seconds, the culture was mixed with approximately 0.7× volume of ice and harvested by centrifugation. The cell pellet was washed using breaking buffer [49], suspended with breaking buffer containing 1% Triton X-100, and disrupted by vortexing with glass beads. Cell debris were removed by centrifugation at 20 kg for 10 min, the supernatant was layered on 5% buffered sucrose, and ribosomes were pelleted as 34000 rpm for 2 h in an SW60 Ti rotor (Beckman, CA USA). The last pelleting step was repeated, and the ribosome fraction was used for RNase protection assays. Thirty OD_260_ units of a ribosome sample was treated with 360 units of RNaseI (Epicentre, Madison USA) or 60 units of MNase (Takara Bio, Tokyo Japan) for 60 min at 37°C. The RNase-treated samples were centrifuged through a 5–45% sucrose gradient for 2 h at 34000 rpm using a SW60 Ti rotor. The typical pattern of the ribosome, with or without RNase treatment, after the sucrose gradient is shown in Additional file 1: Figure S4. The monosome fraction was harvested, and RNA was isolated as described in the previous section. RNA fragments 30–70 nts long were purified using acrylamide gel electrophoresis under denaturing conditions.

### Library construction and DNA sequencing

A cDNA library designed for Illumina sequencing was constructed as described by Ingolia [17]. The libraries were quantified using an Agilent Bioanalyzer 2100 (Agilent, Santa Clara, CA, USA) and sequenced from the 5′ end using single-end sequencing with a Genome Analyzer IIx (Illumina, San Diego, CA, USA).

### Genome mapping of sequence reads

The 3′ adapter and poly(A) sequences were removed from each sequence read using fastx_clipper in the FASTX-Toolkit (http://hannonlab.cshl.edu/fastx_toolkit/index.html) and mapped to the sequence of the *E. coli* K-12 MG1655 genome (GenBank: U00096.2) using bowtie [50].

### Gene-level depth calculation from RP and mRNA-seq data

Based on the gene annotation [26], the number of reads that contained at least a part of each gene was counted, and the sum of the coding sequence (CDS) reads was determined (Additional file 1: Table S3). The normalized depth (RPKMc : reads per kilobase million CDS reads) of each gene was calculated as follows: (number of reads of the gene)/(length of the gene in 1000 nts)/(total CDS reads)× 10^6^. For RP data three samples of BW25113, one MNase-treated and two RNaseI-treated samples, and one sample of BW25113 *smpB*, treated by RNaseI, were used. For mRNA-seq, two samples from BW25113 and one from BW25113 *smpB* were used. An average 3.6 × 10^6^, and minimum >1 × 10^6^, reads for a sample were mapped to the coding genes.

### Nucleotide-level mapping and calculation of local ribosome density

To compare the read depth of each genome position (Figure 2), mapping information of each read was used to summarize the read depth at each genome position of each strand, and the data were normalized between samples by defining the average depth of the total coding region as one. For more precise mapping of ribosomes on mRNA, positions corresponding to the 3′ end of each read were mapped on the genome to calculate the depth of each position of each strand. The relative depth of a genome position was calculated by normalizing the depth on the position according to the average depth from -60 to +60 nts. Because the depth varied among the three bases of the codon, it was normalized using the average of all codons of all genes, excluding the first 20 and last 10 codons where ribosome depth was particularly high. RNaseI-treated samples were used for this analysis. It is not possible to discriminate an A residue at the RNA 3′-end that is protected from nuclease digestion versus the residues added by poly(A)-tailing. Therefore, we compared the ribosome density at each A-site codon between the results using all sequence reads and those using sequence reads that were not affected by this issue (removing reads with 3′-ends mapped to a position followed by A). We found that the difference was minor and became less when usage of the codon became greater (Additional file 1: Figure S5), indicating that a greater number of reads yielded better results, even considering this issue. Therefore, we decided to include all reads for codon-level mapping.

### Informatics

SAM analysis was performed using MultiExperiment Viewer package (http://www.tm4.org/mev.html), DAVID was performed using the website (http://david.abcc.ncifcrf.gov), and statistical analyses were conducted by the R environment [51].

### Data availability

Primary sequence data set for all ribosome profiling and mRNA-seq was deposited in the DNA Data Bank of Japan as BioProject ID PRJDB2960.

CAI for each gene was calculated using the G-language [52] and the gene annotation by Riley et al. [26]. The init-score was calculated using the RBS calculator [30] (https://salis.psu.edu/software/).

## Electronic supplementary material

Additional file 1: **Figures S1 to S5 and Tables S1 to S3.** (PDF 508 KB)

## Abbreviations

RP: Ribosome profiling
PCR: Polymerase chain reaction
RBS: Ribosome binding site
CDS: Coding sequences
RPKMc: Reads per kilobase million CDS reads
R/m ratio: Ratio of RP to mRNA-seq
SAM: Significant analysis of microarray
GFP: Green fluorescent protein
CAI: Codon adaptation index
P-site: Peptidyl site
A-site: Aminoacyl site
E-site: Exit site
Cm: Chloramphenicol
Tet: Tetracycline
nts: Nucleotides
SD: Shine–Dalgarno
PTC: Peptidyl transferase center.
