# Supplementary material for: Effect of codon adaptation on codon-level and gene-level translation efficiency in vivo
Source: BMC Genomics. 2014 Dec 16;15(1):1115. doi: 10.1186/1471-2164-15-1115 (PMC4378010; doi:10.1186/1471-2164-15-1115)
Supplement: Supplementary file 1 — Additional file 1: Figures S1 to S5 and Tables S1 to S3. (PDF 508 KB) [file 12864_2014_6822_MOESM1_ESM.pdf]

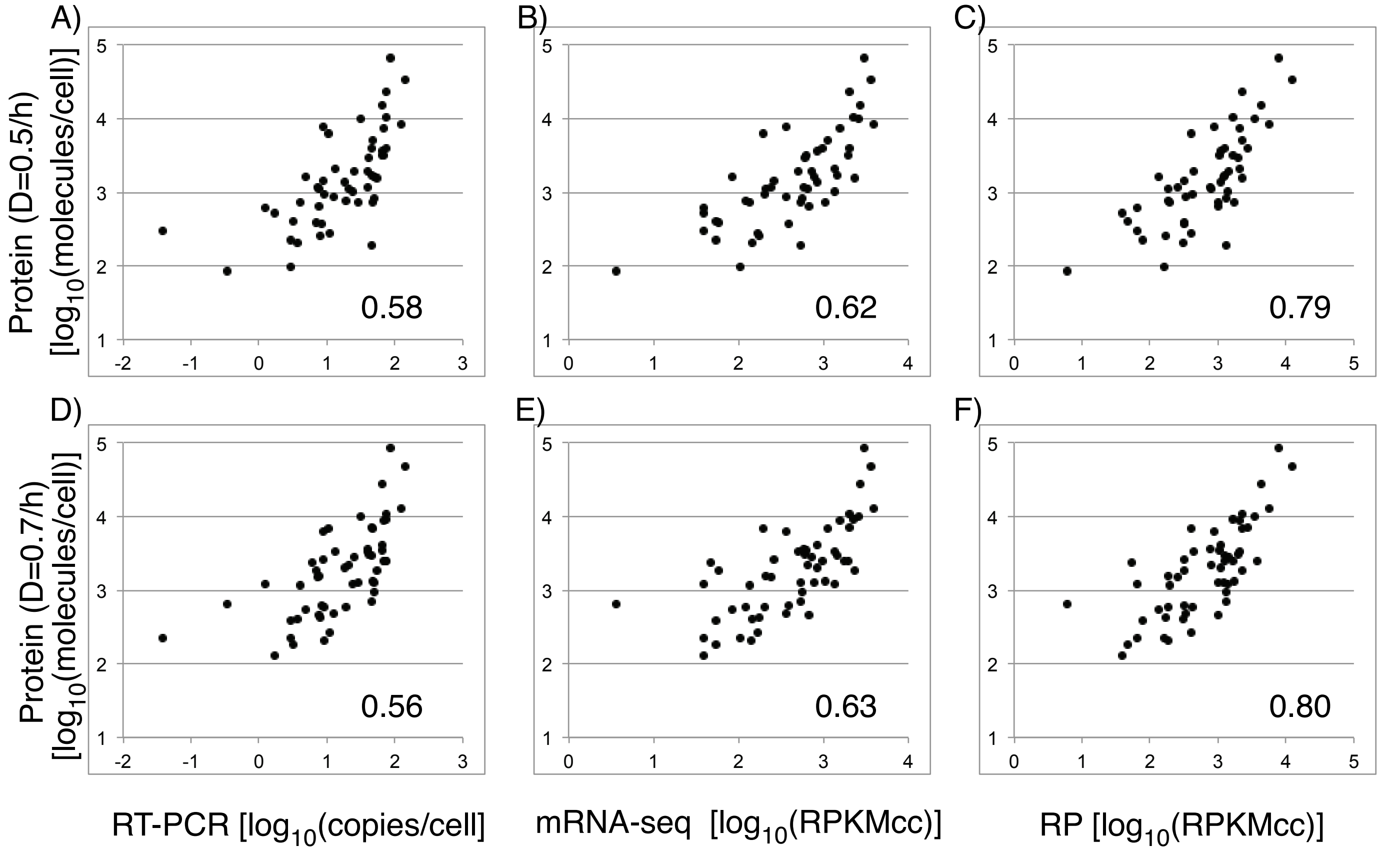

Figure S1. Protein abundance compared with mRNA-seq and ribosome profile data

Three results of RNA quantification among 55 genes were compared with the amount of the corresponding protein, and their correlation coefficients are shown. RT-PCR (D = 0.7 h^{-1}) and protein quantification data (D = 0.5 and 0.7 h^{-1}) are as those from Ishii et al., and the values were converted to molecule/cell. RPKMcc for the genes analyzed using mRNA-seq and RP (D = 0.6 h^{-1}) are those of the present study.

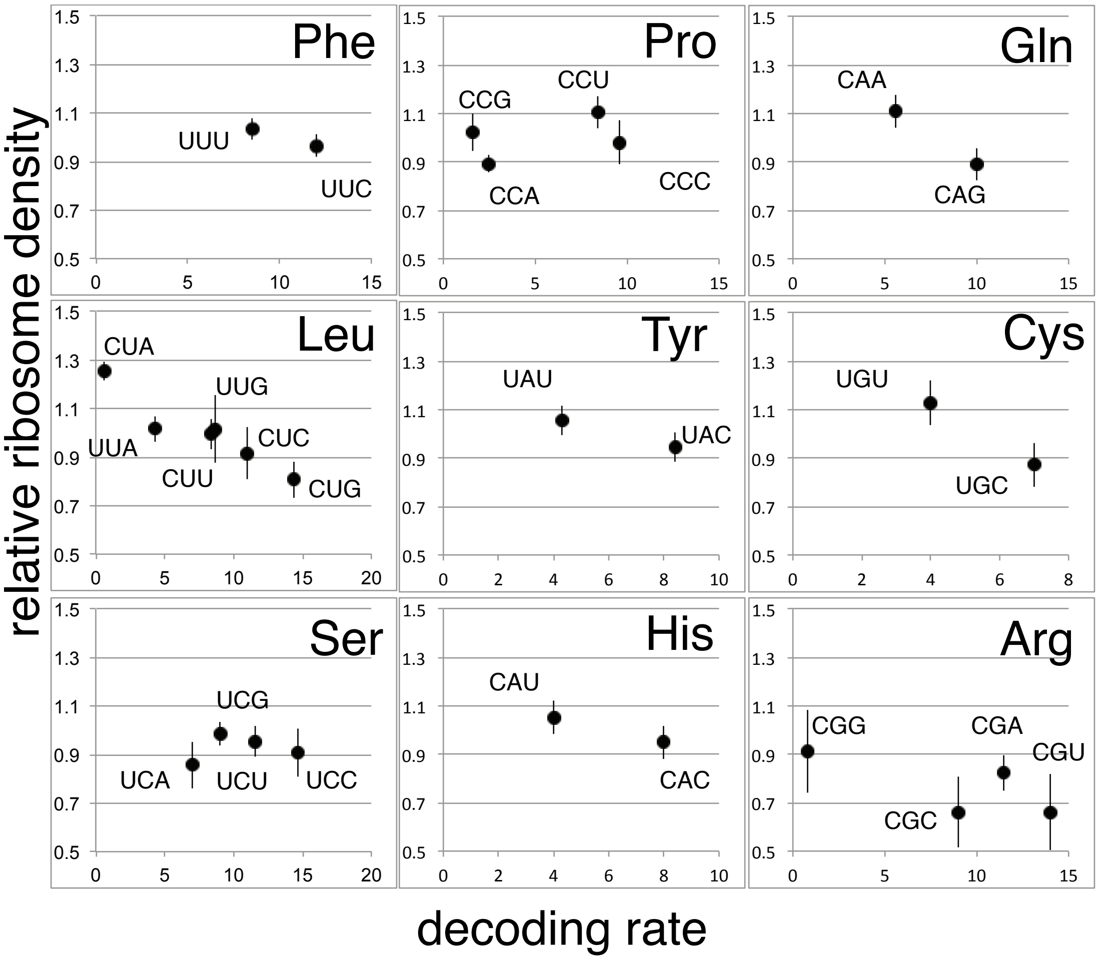

Figure S2. A-site ribosome density compared with decoding rate

The relative A-site ribosome density (shown in Figure 5, Y-axes) was compared with the RtRNA/Rshift value (decoding rate, Curran and Yarus [10], X-axes) . Only YNN codons that could be analyzed using their system are shown.

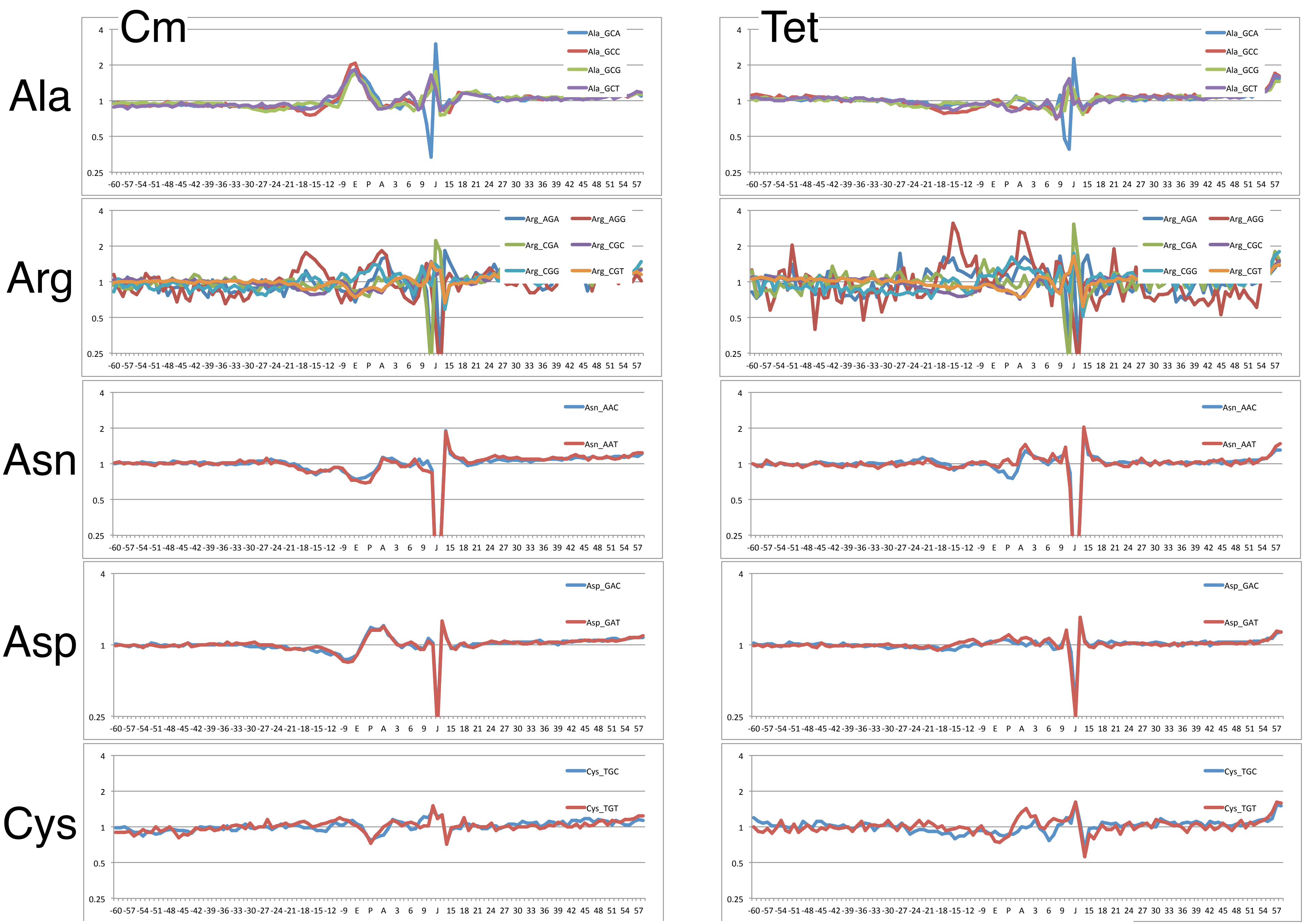

Figure S3 (1/4) Relative ribosome density of each codon at different locations.

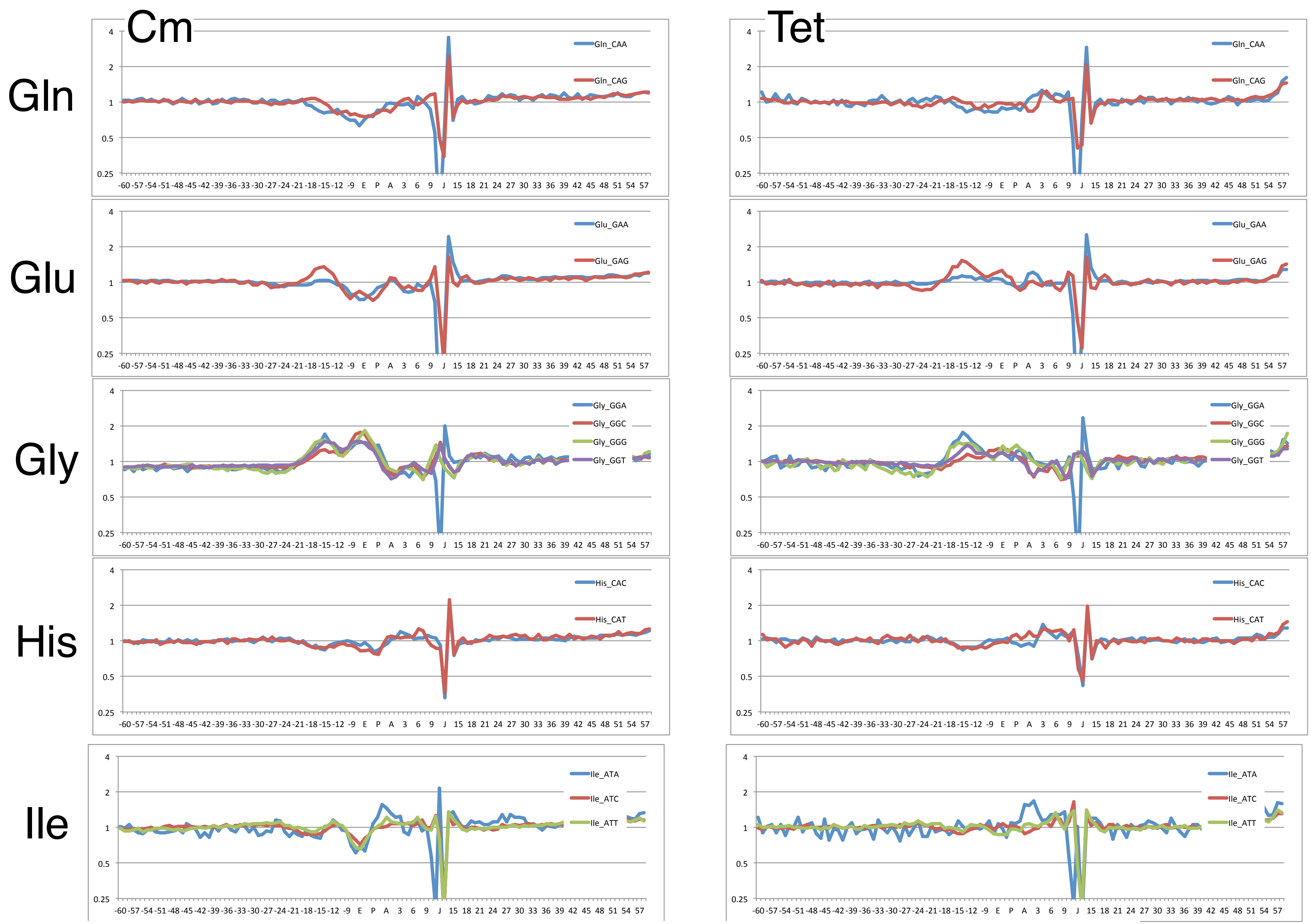

Figure S3 (2/4) Relative ribosome density of each codon at different locations.

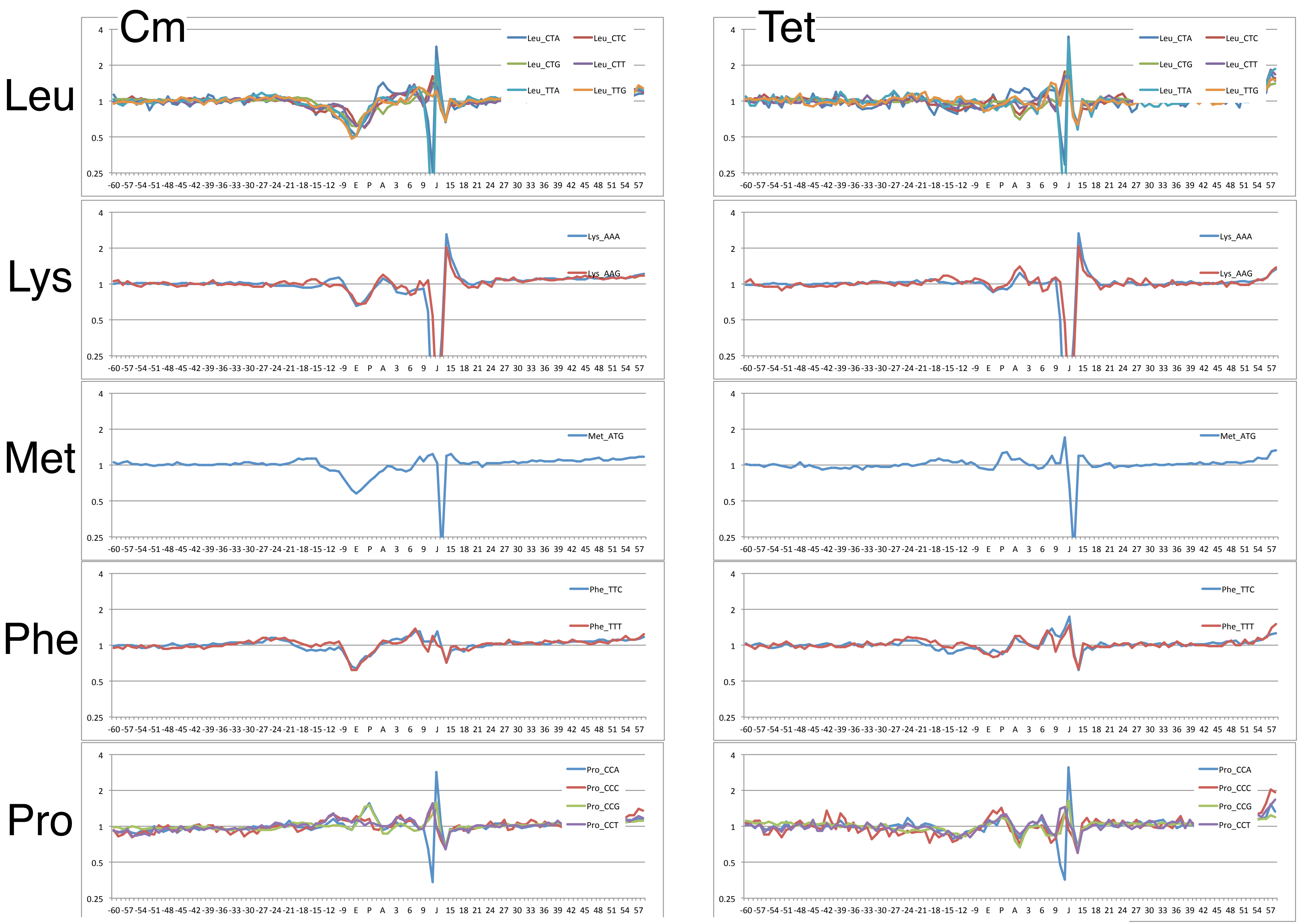

Figure S3 (3/4) Relative ribosome density of each codon at different locations.

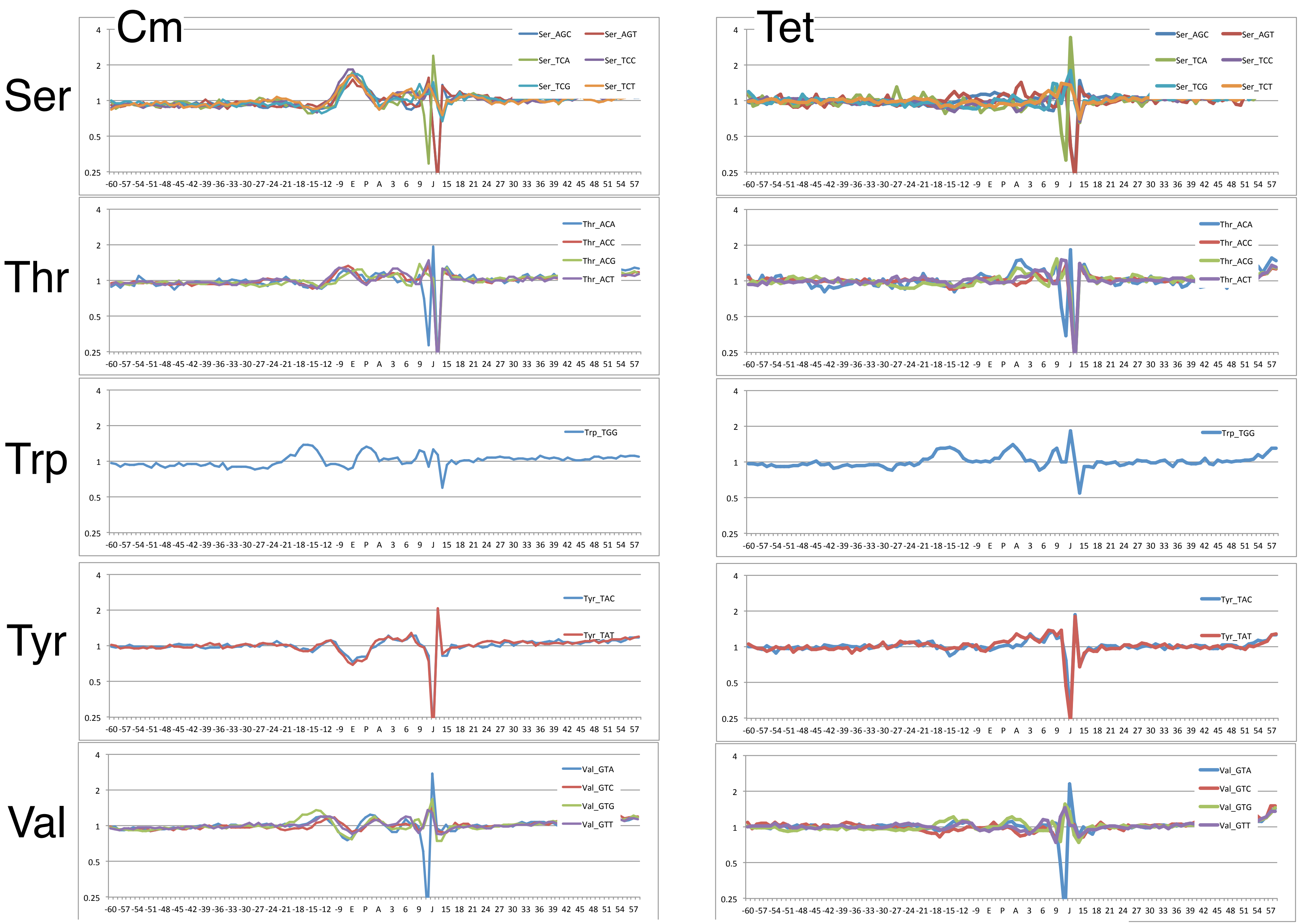

Figure S3 (4/4) Relative ribosome density of each codon at different locations.

Figure S3. Local ribosome densities of codons located at each position

Local ribosome density of each of 61 in-frame coding codons when that is located at each position was shown. One panel shows codons encoding an amino acid and samples taken from cultures treated with Cm or Tet. The x-axis shows the nucleotide position, and the first position of codon at A-site is designated as zero. Positions corresponding to ribosomal A, P, and E-site and the 3′ end of the sequence read (J) are marked.

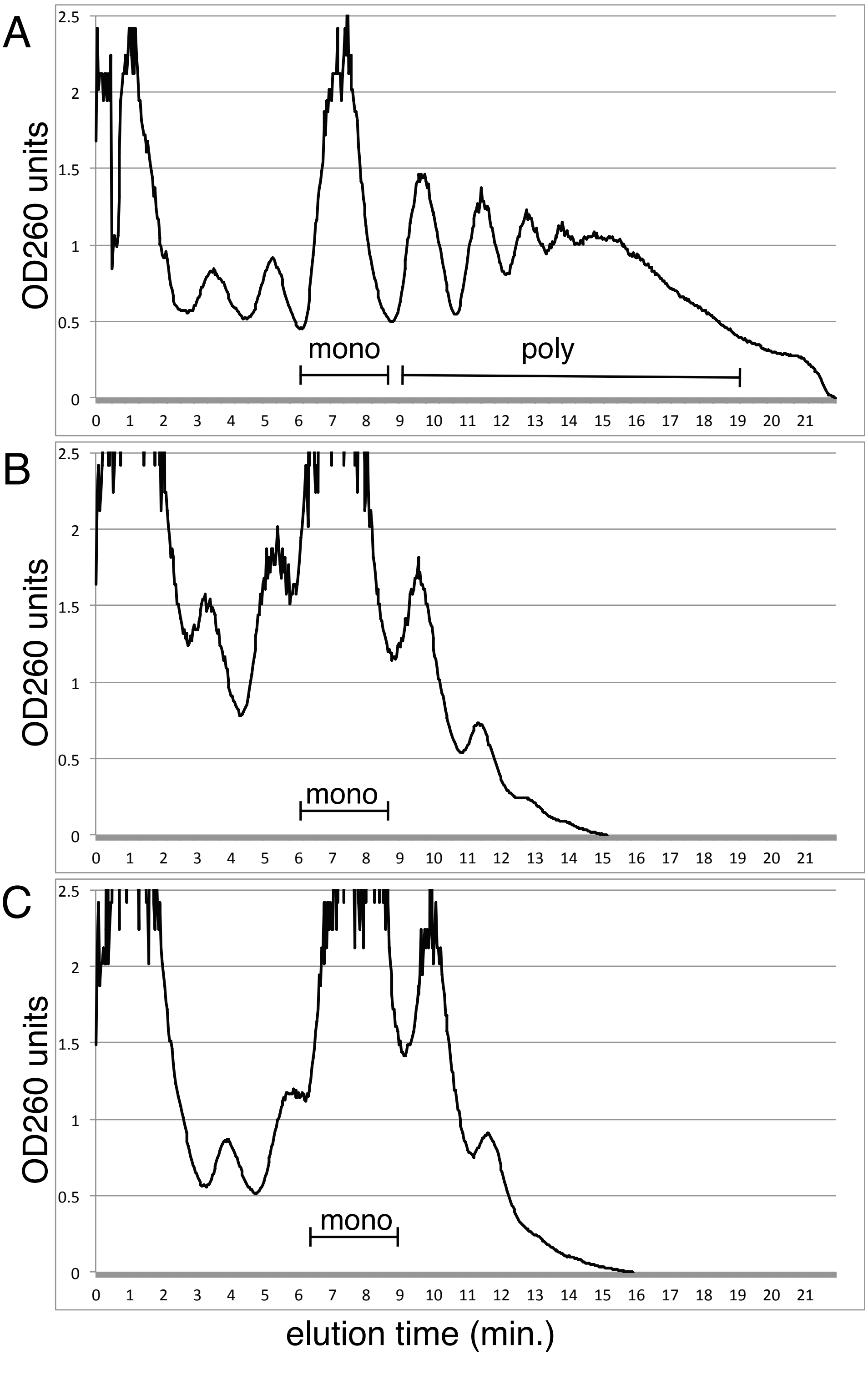

Figure S4. Sucrose density gradient pattern of ribosome fraction

Sucrose density gradient pattern of ribosome fraction without RNase treatment (A), or treated by RNaseI (B) or MNase (C). After the sucrose gradient centrifugation, solution was eluted from the top (light) and OD260 was monitored. Monosome (mono) and polysome (poly) sections were indicated.

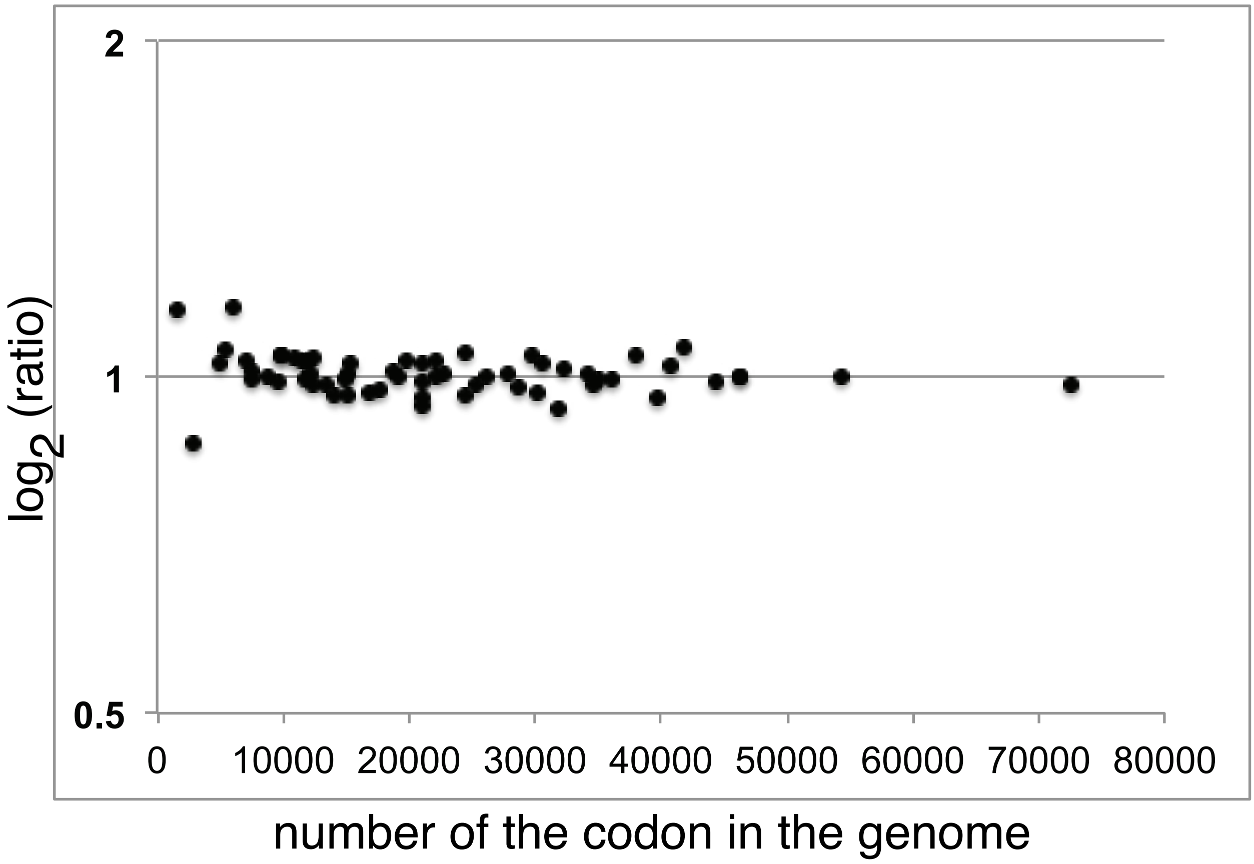

Figure S5. Ribosome density of each codon at A-site was calculated using all the sequence reads or reads whose 3′-end was not mapped to the position followed by A. Then the ratio of the two results for a codon was plotted against usage of the codon in the genome.

Table S1 RNA and protein abundance

Gene	RNA (qRT-PCR, Ishii et al, D=0.7/h, molecule/cell)*1	RNA (mRNA-seq, DPB, D=0.6/h)*2	RNA(RP, DPB, D=0.6/h)*2	protein(molecule/cell, ishii et al, D=0.5/h)*1	protein(molecule/cell, ishii et al, D=0.7/h)*1
aceA	73.57	2011.1	2268.6	23607.3	11076.0
aceB	73.81	2226.0	1627.6	10445.3	9376.7
aceE	69.88	1944.9	1626.7	3212.6	2490.8
aceF	23.53	1375.2	1390.2	1015.0	1193.4
ackA	10.75	192.4	408.8	6253.7	6899.4
acnA	9.22	143.1	189.8	ND *3	209.7
acnB	48.77	559.9	1311.5	834.9	938.2
acs	6.02	46.8	54.6	ND	2399.7
adhE	46.35	534.8	1333.6	192.1	705.6
adk	47.70	1030.0	1749.0	719.2	1339.9
eda	8.68	365.6	874.8	7629.5	6396.5
eno	66.38	2685.6	4290.7	15018.2	27470.3
fbaA	46.20	2029.0	2787.7	3929.3	7244.8
fbp	7.18	239.4	254.0	1152.2	1472.6
frdA	1.73	39.6	38.4	531.3	129.9
fumA	19.15	121.6	183.2	758.5	588.9
fumB	0.35	3.7	6.1	85.9	656.9
fumC	2.98	54.4	78.1	223.1	387.6
galM	8.27	386.1	318.3	371.9	616.7
gapA	143.33	3693.2	12608.2	33987.3	47941.4
glcB	3.26	54.6	46.5	397.3	181.7
gltA	75.85	991.6	1284.2	3870.6	2494.3
gnd	53.37	2305.5	2267.0	1593.2	1852.3
gpmA	31.80	2650.2	3411.7	9979.3	10017.4
icd	122.67	3943.4	5741.2	8619.3	12662.7
ldhA	4.00	137.0	194.1	716.8	1163.4
lldD	1.25	38.9	64.6	630.1	1233.9
lpd	67.61	1574.7	2074.7	7291.0	8810.4
mdh	73.01	1755.4	3783.5	ND	2440.9
pck	4.81	84.6	136.9	1650.8	533.9
pfkB	6.96	57.7	325.5	385.8	1880.1
pflA	2.94	103.8	162.1	96.5	224.7
pflB	49.08	796.6	1185.2	1647.1	1291.0
pgi	20.49	650.4	796.6	1097.5	2164.9
pgk	46.83	1126.6	2337.3	5043.8	6850.6
pgm	12.47	358.1	328.5	861.4	473.1
ppc	40.05	581.9	767.0	1152.3	3597.3
pps	8.08	175.1	169.3	258.7	418.3
prpC	0.04	38.9	64.6	297.4	222.5
pta	3.63	148.1	311.1	206.1	398.5
ptsH	85.61	3005.3	7825.4	66387.7	86935.8
ptsI	44.94	1458.7	1247.4	1686.8	2992.0
pykA	9.06	200.6	427.3	943.7	584.0
pykF	24.91	723.2	1425.3	1958.7	2862.1
rpiA	7.52	681.9	1010.9	633.6	469.2
sdhA	39.26	496.8	439.8	1890.3	3383.7
sucB	65.25	624.7	1031.6	3214.0	3438.2
sucC	65.32	846.4	1125.4	3589.7	4206.5
sucD	18.06	833.5	1092.1	1387.3	1977.7
talA	8.68	259.7	318.6	1425.0	2563.0
talB	41.09	616.9	2016.6	2984.4	3127.9
tktA	28.29	543.9	1011.5	726.8	1265.0
tktB	7.48	212.0	184.5	1136.0	1574.2
tpiA	13.22	1341.5	2077.8	2136.0	3304.8
ybhE	11.13	171.2	395.3	281.1	270.2

**1* : Ishii N, et al. 2007. *Science* 316(5824): 593-597.

**2* : this work

**3* : not detected

Table S2 Read depth (DPB) and R/m ratio of the SAM selected genes.

gene	R/m-ratio *1	Expected score (dExp) *2	Observed score(d)*2	Numerator(r)*2	Denominator (s+s0)*2	RP(GAII5_3)*2	RP(GAII5_5)*3	RP(GAII9_4)*3	RP(GAII9_2)*3	mRNA(GAII5_6)*3	mRNA(GAII6_1)*3	mRNA(GAII8_10)*3
rplY	3.447	−0.036	−4.736	−3.417	0.722	12.089	12.292	12.672	12.984	9.065	8.877	9.333
rpsF	3.507	0.953	−4.403	−3.495	0.794	13.084	13.281	12.672	12.267	8.910	9.354	9.731
speG	2.627	−0.248	−4.206	−2.616	0.622	8.534	8.341	7.970	8.122	5.468	5.695	5.716
rpsR	2.838	0.959	−4.163	−2.829	0.680	12.785	12.881	12.284	12.336	9.522	9.686	10.020
pheL	3.367	0.100	−4.141	−3.342	0.807	14.834	14.450	15.378	15.507	11.676	12.101	11.325
uspA	2.847	0.462	−4.023	−2.818	0.700	12.233	11.997	12.049	11.355	9.124	8.917	9.232
phnA	2.790	0.846	−3.839	−2.851	0.743	8.977	8.605	8.912	8.657	5.586	6.555	5.669
ygfZ	2.355	0.223	−3.656	−2.360	0.646	9.163	9.189	8.792	8.811	6.373	6.594	6.918
ftn	2.548	−0.131	−3.498	−2.557	0.731	8.161	7.387	7.639	7.763	4.841	5.613	5.087
pyrC	1.943	−0.456	−3.481	−1.951	0.560	9.817	9.864	9.938	9.916	7.796	7.847	8.155
asnC	2.451	0.582	−3.423	−2.415	0.706	6.222	5.893	5.394	5.443	3.243	3.225	3.500
deoC	2.189	1.490	−3.410	−2.181	0.640	7.906	7.700	7.441	7.348	5.194	5.476	5.582
ydiH	2.317	−0.214	−3.346	−2.289	0.684	10.727	9.862	10.217	10.244	7.982	8.123	7.816
rplI	2.434	0.960	−3.300	−2.437	0.739	12.160	12.505	12.250	11.673	9.319	9.724	10.087
pfkB	2.417	−0.195	−3.175	−2.495	0.786	8.328	8.470	8.468	8.118	6.262	5.113	6.179
yadD	−2.008	−1.093	2.642	2.043	0.773	2.610	3.206	3.147	3.755	5.568	5.056	5.046
cdaR	−1.912	−1.043	2.657	1.951	0.734	2.145	2.125	1.508	2.566	4.100	4.185	3.826
yjiE	−1.743	1.219	2.660	1.765	0.664	3.374	3.607	4.090	3.942	5.553	5.633	5.370
sfsA	−1.633	−1.070	2.661	1.634	0.614	5.500	5.659	5.324	5.790	7.208	7.001	7.397
norW	−2.177	0.143	2.670	2.214	0.829	−0.526	0.418	0.316	−0.574	1.715	2.505	2.148
ypjB	−2.438	0.123	2.683	2.548	0.950		−1.649		−0.378	2.233	2.226	1.666
hcaE	−2.409	0.078	2.684	2.502	0.932	1.910	2.093	0.789	2.512	3.911	4.622	4.453
rimK	−1.648	−0.535	2.696	1.657	0.615	5.648	5.546	5.126	5.358	7.131	7.188	6.910
ypfI	−1.988	0.056	2.702	2.055	0.761	3.814	3.861	3.708	4.778	6.177	5.952	6.158
rem	−2.569	−0.257	2.714	2.636	0.971	2.644	2.003	0.901	1.596	4.885	3.800	4.581
ugd	−1.806	−0.088	2.719	1.836	0.675	2.892	2.921	3.548	3.489	5.155	5.004	4.988
ycjM	−1.921	−0.352	2.728	1.957	0.717	−1.093	−1.149	−1.836	−0.878	0.885	0.594	0.674
ycfN	−2.396	−0.437	2.732	2.587	0.947	3.577	3.847	4.048	5.409	6.875	6.879	6.669
creB	−1.955	1.869	2.734	1.935	0.708	4.835	5.135	4.770	5.321	7.355	6.942	6.554
topB	−1.687	−0.181	2.740	1.698	0.620	5.230	5.605	5.244	5.701	7.033	7.295	7.101
recB	−2.321	0.192	2.744	2.445	0.891	2.586	3.666	3.463	4.328	6.105	5.880	5.881
ykgL	−2.929	−0.854	2.756	3.487	1.265		0.148		2.741	4.445	4.167	4.015
dusB	−2.595	0.353	2.757	2.884	1.046	5.447	6.025	5.704	7.641	9.031	9.167	9.065
djlC	−2.279	−0.617	2.757	2.397	0.869	1.818	0.647	0.375	1.503	3.474	3.579	3.393
yjjY	−2.955	2.237	2.758	3.198	1.159				2.112	4.460	3.468	3.249
yfjL	−2.058	0.115	2.763	2.123	0.768	3.050	3.643	3.774	4.300	5.895	5.837	5.713
yegK	−2.439	−0.072	2.784	2.510	0.902	−0.952	−1.593	−0.695	0.000	1.704	2.121	1.274
gntX	−3.519	0.428	2.785	4.328	1.554	1.526	1.563	−0.540	3.942	5.837	6.282	5.732
yraN	−2.166	0.307	2.799	2.251	0.804	5.037	4.159	4.571	5.492	6.988	7.128	7.081
hyfR	−2.680	0.062	2.800	2.848	1.017	−1.354	−1.410	−2.097	−0.023	1.887	1.880	1.114
truB	−1.739	0.316	2.828	1.754	0.620	6.050	6.330	6.123	6.618	7.993	8.156	7.954
yphB	−1.512	0.080	2.839	1.515	0.534	2.437	2.298	2.430	2.182	3.830	3.860	3.867
ydcM	−1.910	−0.308	2.879	1.916	0.665	4.704	4.881	5.339	4.857	7.004	7.015	6.565
yadB	−1.786	−1.078	2.893	1.798	0.622	4.087	4.212	3.666	4.176	5.913	5.896	5.692
lpxB	−2.261	−1.008	2.906	2.355	0.810	5.142	5.786	5.342	6.469	8.042	8.097	7.979
ylbF	−2.251	−0.689	2.913	2.269	0.779		−0.692		0.386	2.605	1.935	2.038
ydaY	−2.880	−0.328	2.927	3.080	1.052		1.074	0.386	−1.240	3.371	2.816	3.219
mhpC	−2.399	−0.802	2.929	2.485	0.848	3.085	2.655	1.901	3.349	5.017	5.258	5.424
nudB	−2.573	−0.143	2.936	2.745	0.935	5.322	6.064	5.447	7.057	8.639	8.874	8.638
menE	−1.875	−0.017	2.938	1.886	0.642	4.406	4.396	3.831	4.177	6.022	6.287	5.955
ybiW	−1.730	−0.543	2.949	1.735	0.588	0.180	0.317	0.215	0.588	2.029	1.944	2.206
ycjT	−2.813	−0.350	2.990	3.054	1.022	−1.526	−1.582	−0.684	0.426	2.378	2.161	2.099
yahM	−4.210	−0.820	2.995	4.302	1.436	3.001	2.623		2.309	6.761	7.124	4.970
rimM	−2.714	0.104	3.016	2.921	0.968	8.881	8.954	8.297	10.317	11.988	11.997	12.112
yehM	−2.567	−0.057	3.027	2.710	0.895	−2.533	−1.589	−2.276	−0.903	0.930	0.845	0.878
astB	−2.561	−0.186	3.029	2.663	0.879	1.037	−0.090	−0.514	0.444	3.055	2.573	3.019
tpr	−4.169	−0.386	3.033	5.183	1.709		2.308		4.279	7.249	7.001	6.240
rpoE	−2.232	0.091	3.059	2.291	0.749	7.124	7.403	6.928	8.021	9.534	9.682	9.763
yjjN	−1.992	1.349	3.067	1.982	0.646	−0.377		−0.120	−0.425	2.086	1.609	1.560
rpoA	−1.643	0.368	3.074	1.648	0.536	11.253	11.390	11.434	11.559	13.059	13.059	13.052
yghO	−2.726	0.253	3.085	2.871	0.931	−1.574	−1.215		−0.137	2.252	2.219	1.948
ykiA	−2.513	−0.776	3.086	2.595	0.841	3.652	2.841	2.324	3.504	5.782	5.826	5.419
ybeQ	−2.377	−0.620	3.098	2.447	0.790	0.273	−0.953		0.125	2.344	2.318	2.262
ynaK	−2.838	−0.329	3.103	2.866	0.924		0.936		1.207	2.818	3.955	3.432
yhcG	−2.336	0.340	3.134	2.353	0.751	0.482	0.841	0.739	1.434	3.598	2.927	3.156
ychA	−1.712	−0.392	3.135	1.717	0.548	6.189	6.319	6.159	6.466	7.947	8.042	8.010
ycjS	−3.115	−0.351	3.160	3.345	1.059	0.162	−2.064	−1.166	−0.208	2.693	2.371	2.514
ydhW	−2.652	−0.220	3.164	2.792	0.882		−0.359	−0.462	1.082	2.938	2.727	2.907
yfaE	−2.211	−0.025	3.170	2.250	0.710	4.949	5.234	4.884	5.765	7.623	7.327	7.422
xylG	−3.023	0.486	3.202	3.261	1.019	−1.969	−1.025	−1.712	0.120	2.272	2.104	1.968
aspA	−2.601	0.877	3.210	2.671	0.832	3.381	4.286	4.747	4.504	7.164	6.706	6.832
ycaQ	−3.140	−0.508	3.240	3.434	1.060	0.354	1.035	0.610	2.598	4.682	4.634	4.434
holA	−2.024	−0.623	3.261	2.040	0.625	4.985	5.025	5.171	5.562	7.343	7.202	7.132
truA	−2.164	0.006	3.263	2.196	0.673	5.183	5.347	5.189	5.920	7.687	7.632	7.496
hydN	−2.501	0.144	3.277	2.553	0.779		0.936		0.792	2.818	2.885	3.251
aceK	−3.133	0.767	3.278	3.312	1.010	3.836	4.993	5.411	5.981	8.436	8.106	8.559
intR	−3.087	−0.339	3.291	3.295	1.001	0.935	0.709	0.929	2.595	4.707	4.828	4.226
mraZ	−2.496	−1.209	3.303	2.541	0.769	6.521	6.789	6.265	7.372	9.580	9.139	9.113
yagJ	−2.816	−0.876	3.355	2.957	0.881	1.106	1.272	0.948	2.491	4.285	4.432	4.517
yegZ	−2.949	−0.068	3.360	2.943	0.876		0.206		1.062	3.825	2.640	3.314
yjeN	−3.017	0.894	3.361	3.170	0.943				1.537	3.786	3.701	3.177
hofB	−3.167	−1.154	3.397	3.288	0.968	−0.230		−1.558	0.274	3.163	3.029	2.537
yggD	−2.716	0.235	3.413	2.779	0.814	3.949	3.741	4.528	4.958	7.306	7.141	6.774
smf	−2.509	0.361	3.531	2.558	0.724	3.843	4.271	3.872	4.774	6.626	6.776	6.842
yghF	−2.351	0.249	3.552	2.348	0.661	1.447	1.221	1.440	1.814	4.103	3.848	3.536
dnaG	−2.721	0.280	3.646	2.784	0.763	5.032	5.165	5.207	6.107	8.325	8.168	7.991
yicR	−4.601	0.526	3.657	5.018	1.372	2.558	2.180	−0.508	2.820	6.880	6.623	6.839
dnaE	−2.094	−1.003	3.660	2.099	0.573	4.959	5.148	4.939	5.291	7.265	7.225	7.059
ruvC	−2.997	−0.145	3.672	3.120	0.850	4.879	5.040	5.060	6.290	8.526	8.419	8.368
yjiV	−2.998	1.275	3.706	3.103	0.837	0.671	0.893	1.169	2.089	4.407	4.134	4.384
fdhF	−2.474	0.814	3.754	2.497	0.665	2.308	2.234	1.617	1.855	4.359	4.637	4.505
yceD	−2.522	−0.444	3.787	2.549	0.673	9.479	9.938	9.223	9.905	12.177	12.306	12.075
xylA	−2.888	0.485	3.911	2.955	0.756	1.422	1.611	1.316	2.405	4.715	4.560	4.656
yffM	−2.264	0.043	4.016	2.267	0.564		0.038		0.309	2.335	2.472	2.253
mbhA	−3.010	−0.933	4.225	3.011	0.713	−0.941	−0.582	−0.684	−1.311	2.300	1.746	2.350
ybaJ	−3.142	−0.730	4.280	3.165	0.739	5.428	5.015	5.882	5.058	8.327	8.853	8.352
trmD	−4.096	0.103	4.346	4.339	0.998	7.008	7.448	6.937	8.889	11.930	11.926	11.873
mokB	−5.955	−0.312	6.273	5.964	0.951	2.814	2.588	1.486	2.859	8.792	8.743	7.669
yjhD	−4.741	1.097	6.307	4.712	0.747				−0.600	4.426	5.078	4.181

*1: R/m ratio in logalithm base 2, *2: the results from SAM analysis, *3: DPB of the gene in each sample (logalithm base 2) sample names are shown in Table S3.

Table S3 Sequenced samples and number of reads mapped to CDS

Name	source strain	library[*1]	CDS reads[*2]
GAII05_3	BW25113 *smpB*	RP(Cm)	2,538,972
GAII05_5	BW25113	RP(Cm)	3,959,115
GAII_9_4	BW25113	RP(Cm)	2,124,985
GAII_9_2	BW25113	RP(Cm)(Mnase)	6,562,793
GAII06_1	BW25113 *smpB*	RNA-seq	5,127,408
GAII05_8	BW25113	RNA-seq	1,611,231
GAII_8_10	BW25113	RNA-seq	5,967,668
GAII05_4	BW25113	RP(Tet)	1,027,370
GAII07_4	BW25113	RP(Tet)	3,357,792
GAII07_5	BW25113	RP(Tet)	4,498,133

*1 Type of library used (RNA-seq or RP), antibiotics used to stop translation (Cm or Tet), and nuclease used for polysome digestion (MNase: if specified, or RNaseI: if not indicated) are shown

*2 Total number of the sequence reads mapped to CDSs
